# A global view of comorbidity in multiple sclerosis: a systematic review with a focus on regional differences, methodology, and clinical implications

**DOI:** 10.1007/s00415-020-10107-y

**Published:** 2020-07-27

**Authors:** Larissa Hauer, Julian Perneczky, Johann Sellner

**Affiliations:** 1 Department of Psychiatry, Psychotherapy and Psychosomatic Medicine, Christian Doppler Medical Center, Paracelsus Medical University, Salzburg, Austria; 2 Department of Neurology, Landesklinikum Mistelbach-Gänserndorf, Liechtensteinstrase 67, 2130 Mistelbach, Austria; 3 Department of Neurology, Klinikum rechts der Isar, Technische Universität München, Munich, Germany; 4 Department of Neurology, Christian Doppler Medical Center, Paracelsus Medical University, Salzburg, Austria

**Keywords:** Multiple sclerosis, Comorbidity, Autoimmunity, Prevalence, Risk factors, Treatment, Quality of life

## Abstract

Multiple sclerosis (MS) is a chronic autoimmune disorder of the central nervous system which is associated with numerous comorbidities. These include cardiovascular disease, psychiatric and neurologic disturbances, restless leg syndrome, migraine, cancer, autoimmune diseases, and metabolic disorders. Comorbid disease is an important consideration for clinicians treating patients with MS; early presentation of comorbidities can obscure or delay MS diagnosis, as well as significantly impacting the disease course. Improved understanding of comorbidities and their emergence in MS populations is important for improving the quality of life and optimizing treatment for patients. Therefore, we evaluated published studies reporting epidemiologic data on comorbidities and their associated impact on disease progression in patients with MS (PwMS). The prevalence of neurologic, cardiovascular, metabolic, and autoimmune comorbidities was elevated in PwMS in general, and furthermore, this adversely affected a broad range of outcomes. Compared with PwMS, cancer rates in people without MS or the general population were lower, which should prompt further studies into the mechanisms of both diseases. Studies were under-represented in many regions owing to the latitudinal gradient of MS and possible underfunding of studies.

**Electronic supplementary material:**

The online version of this article (10.1007/s00415-020-10107-y) contains supplementary material, which is available to authorized users.

## Introduction

Multiple sclerosis (MS) is an immune-mediated chronic disease of the central nervous system (CNS) that affects individuals in their early adult life, and can have drastic consequences on functional, emotional, and financial course of the patient’s life [1]. The disorder is characterized by intermittent, recurrent, and focal episodes of inflammatory demyelination and transection of CNS axons [2, 3]. Environmental, genetic, and epigenetic factors have a causal role in MS and potentially interact with modifiable risk factors [4–7].

Comorbidities across multiple body systems are common in patients with MS (PwMS) and are associated with diminished quality of life (QoL), and long-term disability [8, 9]. Depression, anxiety, cardiovascular disease, epilepsy, metabolic disease, and autoimmune diseases are the most common comorbidities in PwMS [10–13].

Comorbid diseases are a critical issue for clinicians treating PwMS as they can adversely affect a broad range of outcomes in MS, including the risk of relapse and disease progression [14]. While comorbidities are a clear concern throughout the disease course for PwMS, some comorbidities may also present very early in the disease course, which can obscure or delay MS diagnosis [8, 15]. Therefore, a multidisciplinary approach and timely recognition of comorbid conditions are paramount to initiating effective treatment in PwMS [16–18].

Understanding the global impact of comorbidities on PwMS is challenging for several reasons. MS is not evenly distributed worldwide; instead, the disease prevalence shows a latitudinal gradient [19]. Furthermore, studies investigating comorbidities in PwMS have typically been performed in small cohorts making regional comparisons challenging. Understanding the epidemiology of comorbidities and their impact on MS progression is important for optimal treatment of MS and for improving the QoL for PwMS. Therefore, a clear understanding of the risk of developing these conditions and their prevalence in established MS populations is needed. In this review, we present recent studies investigating MS comorbidities and their impact on MS prognosis, noting where potential regional differences may be a factor.

## Methods

We searched PubMed for studies on comorbidities in PwMS, using a time-restricted filter (Nov 2009–Nov 2019) and the following terms as determined in the set of systematic reviews by Marrie et al. [10–12, 20–22]:

“multiple sclerosis AND (cohort OR cross-sectional) AND (diabetes OR hypertension OR hyperlipidemia OR hypercholesterolemia OR “ischemic heart disease” OR “valvular disease” OR arrhythmia OR “congestive heart failure” OR “cerebrovascular disease” OR stroke OR “transient ischemic attack” OR “cerebral infarction” OR “cerebral hemorrhage” OR “peripheral vascular disease” OR “autoimmune disease” OR “alopecia areata” OR “ankylosing spondylitis” OR “autoimmune thyroid disease” OR “bullous pemphigoid” OR “celiac disease” OR dermatomyositis OR “idiopathic thrombocytopenic purpura” OR “inflammatory bowel disease” OR “ulcerative colitis” OR “Crohn’s disease” OR “myasthenia gravis” OR “pemphigus vulgaris” OR “pernicious anemia” OR polymyositis OR “primary adrenocortical insufficiency” OR “primary biliary cirrhosis” OR “psoriasis” OR “rheumatoid arthritis” OR “Sjögren’s syndrome” OR “systemic lupus erythematosus” OR “systemic sclerosis” OR uveitis OR vitiligo OR “Wegener’s granulomatosis” OR asthma OR “chronic obstructive pulmonary disease” OR “gallbladder disease” OR “biliary tract disease” OR “gastroesophageal reflux disease” OR “irritable bowel syndrome” OR “liver disease” OR “hepatic disease” OR “peptic ulcer” OR “renal disease” OR “kidney disease” OR cataracts OR glaucoma OR “retinal disease” OR “musculoskeletal disorders” OR “crystal arthropathies” OR fibromyalgia OR osteoarthritis OR epilepsy OR cancer OR “alcohol abuse” OR “alcohol misuse” OR anxiety OR “bipolar disorder” OR depression OR “personality disorder” OR schizophrenia OR psychosis OR “substance abuse” OR “substance misuse” OR migraine OR “systemic venous thrombosis” OR “restless legs syndrome” OR eczema OR “allergic disease” OR “allergic rhinitis” OR anosmia OR hyposmia OR “olfactory dysfunction”).

After eliminating duplicates, we initially screened titles and abstracts, excluding review articles, editorials/comments, articles that were out of the scope of the review or contained no relevant information.

We then performed a secondary screening, of full-text articles, eliminating reports based on the following criteria:

1. The primary measure was prevalence, incidence or risk of MS symptom, including fatigue, cognitive dysfunction, neuropathic or musculoskeletal pain, sleeping disorders, or sexual dysfunction.
2. Treatment goals were the primary objective (e.g., vitamin D, goal adjustment in MS-associated depression).
3. The comorbidity was investigated as an association or the output measure was given as a mean severity score (instead of cut-off incidence/prevalence measure or risk ratio).

## Results

**Fig. 1:**
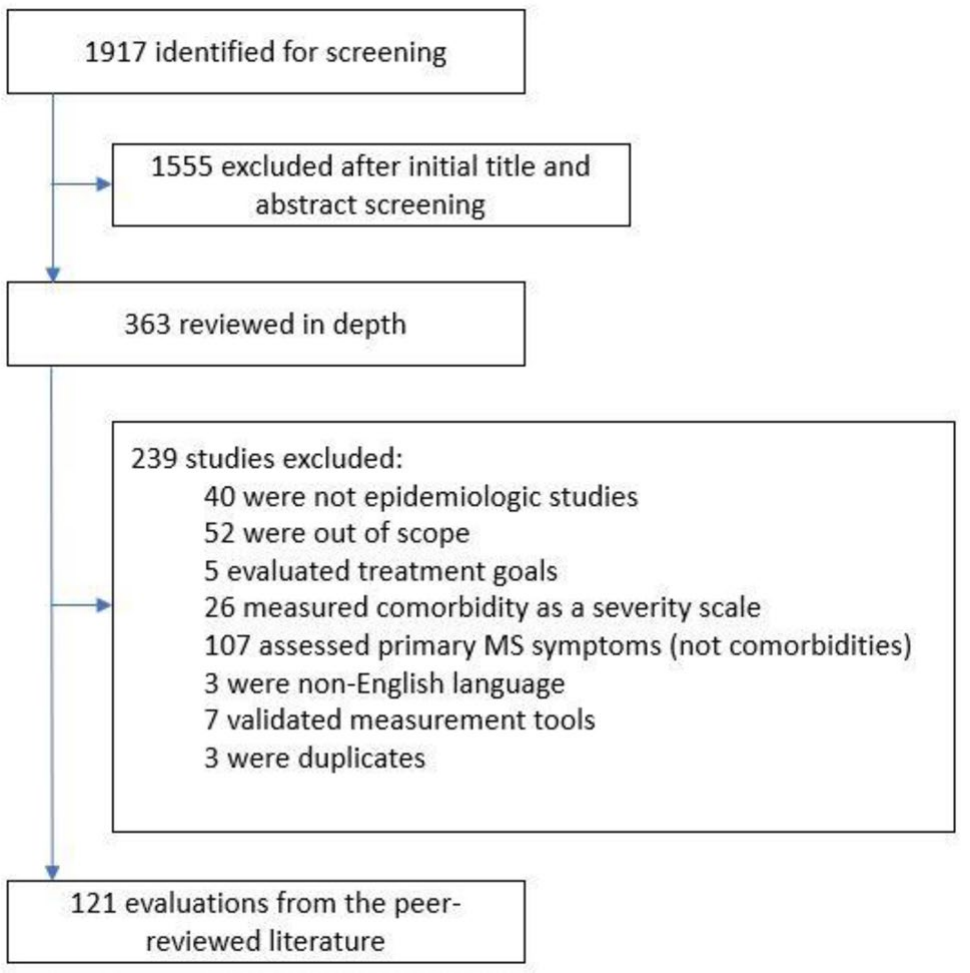
Flowchart of study selection

Our search of peer-reviewed literature yielded 1917 articles, of which 1553 were excluded after title and abstract screening. After reviewing and analyzing 363 articles screening, 121 articles were selected for inclusion (Fig. 1). Prevalence data for individual comorbidities in PwMS are presented in Supplemental Tables 1–9.

### Cardiovascular comorbidities (Supplemental Table 1)

Cardiovascular (CV) comorbidities such as abnormalities in blood pressure (BP) response, heart rate, heart rhythm, and left ventricular systolic function are common in PwMS, and these comorbidities have been reported as the second or third most common cause of death in PwMS [23]. Cardiovascular dysfunction in PwMS often co-occurs with comorbid metabolic conditions, such as diabetes, hypertension, and hyperlipidemia, which are typically elevated in the MS population [11]. While the etiology of CV comorbidity in MS is incompletely understood, and likely underrecognized, Moss et al. [24] suggested that it could be associated with the level of disability, reflecting reduced physical activity levels, greater sedentary behavior, exacerbating weight gain/obesity, and poorer general health. Anemia, while not a cardiovascular abnormality per se, is often noted alongside or associated with cardiovascular disease and, therefore, was included in this group owing to its common occurrence in such complications.

Prevalence studies (*N* = 16) analyzed cohorts from Europe (*n* = 6), the USA and Canada (*n* = 8), and Australia (*n* = 1), countries where MS is highly prevalent, in addition in Taiwan (*n* = 2), where MS is less prevalent [25]. The studies assessing cardiovascular comorbidities in MS included the evaluation of venous thromboembolism, atrial fibrillation, myocardial infarction (MI), acute MI, heart failure, ischemic heart disease and stroke, and these studies were largely performed in controlled population-based cohorts (Supplemental Table 1). No region-specific differences were noted.

All the studies investigating CV comorbidities reported increases in risk, incidence, or prevalence in the cardiovascular morbidities of interest. However, the studies were heterogeneous, precluding comparison of findings, and the possibility of positive reporting bias cannot be excluded. Notably, stroke risk was elevated in the first year compared with subsequent years in two studies [26, 27], highlighting the importance of routinely monitoring for comorbid conditions early in the disease course. Anemic patients were more likely to develop MS [28] or relapse [14], warranting further investigation as a potentially important predictor for MS progression. Notably, comorbid cardiovascular risk factors [BP readings, as well as higher plasma glucose and high-density lipoprotein cholesterol (HDL-C)] were associated with disease-modifying therapy (DMT) use, particularly interferon beta and glatiramer acetate versus natalizumab [29], and the presence of hypertension was associated with changes in brain volume/lesions [30].

### Psychiatric/neurologic comorbidities

Psychiatric comorbidities are a major concern in PwMS; they have been associated with fatigue and reduced QoL, and they may also impact on adherence to DMT. Psychiatric comorbidities are more prevalent in PwMS compared with the general population [10], although the cause and effect are not yet clear. Psychiatric features of MS are a major cause of disability in PwMS, early recognition and a better understanding of how psychiatric comorbidities affect disease progression could help to determine optimal treatment, ensuring better long-term outcomes. For example, psychiatric disturbances in the incipient phase of MS may be predictive of future psychiatric illness in a considerable proportion of PwMS, so their early detection is crucial [31].

#### Depression, anxiety, and bipolar disorder (Supplemental Table 2)

Mood dysfunction, including depression, anxiety, and bipolar disorder, is more common among PwMS than in the general population [10, 32]. Mood disorders are also associated with rapid disease progression and overall decreased QoL among PwMS [14], and can occur before the onset of neurologic symptoms of MS [33].

Depression in PwMS is likely to be, in part, a reaction to physical disability, such as problems with balance and walking, [34, 35] or other defined MS symptoms such as fatigue [36, 37]. Recent studies indicate that an inflammatory component, involving several pro-inflammatory cytokines, such as interferon gamma and tumor necrosis factor alfa may be associated with mood disorders [38] and that structural brain alterations may also play a role [39].

Prevalence studies on psychiatric comorbidities (*N* = 42) examined patients in Europe (*n* = 12), the USA and Canada (*n* = 19), Brazil (*n* = 1), Australia (*n* = 3), the Middle East (*n* = 9) and Taiwan (*n* = 2). These studies (Supplemental Table 2) were reported using numerous different neurologic and diagnostic tools: Athens Insomnia Scale; Beck Anxiety Inventory; Beck Depression Inventory Fast Screen; Beck Depression Inventories; The Center for Epidemiologic Studies—Depression scale; Diffuse Axonal Injury; Expanded Disability Status Scale; Epworth Sleepiness Scale; Fatigue Scale for Motor and Cognitive Functions; Functional Assessment of Multiple Sclerosis; General Health Questionnaire 12; Godin Leisure-Time Exercise Questionnaire; Hospital Anxiety and Depression Scale for depression and anxiety; International Classification of Diseases; Multiple Sclerosis International Quality of Life questionnaire; Numeric Rating Scale; Patient Health Questionnaires; Perceived Stress Scale; Patient-Reported Outcomes Measurement Information System; State-Trait Anxiety Inventory.

The frequency of mood disorders in PwMS is high and significantly elevated when compared with control groups, but prevalence levels vary substantially according to the studied population and the research methods used. Most of the studies were cohort studies without control subjects and overall, global prevalence rates for depression and anxiety ranged from 21.1–59.4% to 28.1–57.0% for depression and anxiety, respectively. Several studies reported depression at ~ 35% [40–49] and anxiety at ~ 55% in PwMS [43–46, 49–52], and exceptionally low rates were observed for studies performed in Abu Dhabi (10.8–17.5% for depression and 4.8–20.0% for anxiety) [53, 54]. Jun-O'Connell et al. reported an increased prevalence of bipolar disorder in PwMS in a cohort from the US (type 1 was more significantly more prevalent than type 2 in the study group: 60% versus 30%, respectively), although this study lacked a control group. Moreover, the majority of these patients reported mood disorders before MS diagnosis, which may have delayed initial consideration of MS [32]. Given that the prevalence of psychiatric conditions is profoundly influenced by geographical location [55], global trends in comorbidity data in PwMS should be interpreted cautiously. For example, anxiety appears to be more common in cultures with European/Anglo roots than others, with the lowest prevalence reported in African cultures, irrespective of MS prevalence [56].

The relationship between different aspects of depression and anxiety may also change throughout the disease course; for example, Hartoonian et al. found that non-somatic symptoms were more strongly associated with anxiety early in the disease and somatic symptoms were more prominently linked to anxiety later in the disease [57]. Some researchers have studied the lifetime prevalence, while others have looked at point prevalence in the course of MS. Although often coexisting, depression and anxiety may be associated with distinct aspects of cognitive impairment and functional outcomes [43, 58]. Point prevalence data lack insight on disease trajectory, which is key to understanding optimal monitoring of PwMS, particularly for those comorbidities that are associated with disability at baseline [59]. Physicians should ideally follow PwMS—not only in exacerbation of psychiatric symptoms but also those who report recent increases in somatic depressive symptoms—as these conditions may forecast an upcoming clinical exacerbation [60], which may be crucial when considering MS prognosis.

#### Epilepsy (Supplemental Table 3)

Epilepsy is more prevalent in PwMS compared with the general population [21]. Although frequently conceptualized as a white matter disease, MS lesions are also present in gray matter, potentially interrupting neuronal circuits. However, it is not clear whether epilepsy is triggered by the initiation of MS or whether MS is a risk factor for developing epilepsy. Structural abnormalities in PwMS, such as cortical demyelinating lesions appearing in early MS [61] or edema surrounding the foci, may be involved [62], but MRI data are non-conclusive [63].

Studies on the prevalence of epilepsy (Supplemental Table 3, *N* = 11) include physician reported, hospital admissions, and administrative health data from Europe (*n* = 5), South America (*n* = 2), Canada (*n* = 1), and Iran (*n* = 2). The reported prevalence of epilepsy in PwMS ranged from 1.9% to 7.6% in MS (~ 3% in 4 studies) and this increased to 8.5% in early-onset MS. Epilepsy and disability were robustly associated, reaching a cumulative incidence of 5.3% in patients with MS with an EDSS score 7 [63], indicating the importance of this comorbidity in relation to prognosis and outlook for PwMS.

It is important to consider that Epilepsy is highest in central Latin America, Chile, North Africa, the Middle East, and Bangladesh, where studies of PwMS and comorbid epilepsy are few or non-existent [64].

#### Restless leg syndrome (RLS) (Supplemental Table 4)

RLS is characterized by an irresistible urge to move the legs and often accompanied by unpleasant sensations and nocturnal occurrence. RLS can severely disturb sleep and QoL of PwMS and is more frequent in PwMS than in the general population [21]. The mechanisms in RLS are not fully understood in PwMS, but may involve aberrant signaling in the dopaminergic system caused by demyelinating or neurodegenerative damage to the diencephalospinal tract [65, 66].

Several cohort studies, cross-sectional studies, and metanalyses have investigated the epidemiology of RLS in PwMS (Supplemental Table 4, *N* = 10). RLS prevalence was found to be elevated in PwMS with an odds ratio of 3–4 in PwMS versus control subjects. In agreement with observations by Minar et al. [66], RLS prevalence varied widely from study to study, ranging from 12.12 to 57.50% in PwMS and 2.5% to 18.3% in controls, one study reported faster MS progression in those patients with concomitant RLS [67]. Supporting the concept of dopaminergic involvement in RLS in PwMS, data from two studies showed that the risk of RSL increased in PwMS with spinal cord lesions [66, 68]. Globally, different regions were well represented across studies, including Europe (*n* = 3), Asia (*n* = 2), the Middle East (*n* = 2), and South America (*n* = 1), and notably, RLS was more prevalent in PwMS outside of Asia (27%) than inside Asia (20%) [65].

### Migraine, fibromyalgia, ocular comorbidities, and olfactory dysfunction (Supplemental Table 5)

Pain is considered one of the most disabling clinical symptoms in patients and can interfere with mobility, employment, and QoL in PwMS. Characterized by widespread musculoskeletal pain, fibromyalgia shares many etiological features with MS, including genetic and environmental factors, in addition to an association with mood disorders and adverse effects on QoL [69]. While neuropathic and musculoskeletal pains represent core symptoms of MS, comorbid headaches (normally migraine) are also highly prevalent in PwMS when compared with the general population. Several theories have been suggested for headache mechanisms in MS, including inflammation-induced brain lesions, altered pain perception [70], DMT-related effects [71], and cortical demyelination in early-onset MS [72].

Other clinical features of MS, such as olfactory disturbances and ocular dysfunction are thought to reflect the complex interaction of inflammation, demyelination and axonal degeneration, as well as lesion loads in brain regions [73–75]. Different ophthalmological manifestations of MS such as optic neuritis, uveitis, and ocular motor involvements have been reported in epidemiological studies and the risk of cataract or glaucoma may be also increased owing to shared mechanisms of disease processes or glucocorticoid treatment.

Studies investigating the epidemiology of migraine fibromyalgia, ocular comorbidities, and olfactory dysfunction in MS (Supplemental Table 5, *N* = 12) included cohort studies, cross-sectional studies, and meta-analyses performed in limited geographic regions (Europe *n* = 4, USA *n* = 4, Canada *n* = 2 and Brazil *n* = 1). Migraine prevalence rates were wide ranging (4.7–49.8%). The observed heterogeneity in prevalence rates was also observed in the meta-analyses (*I*^2^ = 97% *χ*^2^ = 247.10, *P* < 0.00001) [76]. The association between migraine and MS was not shown to be consistent between study groups [70, 76]. While no trend in comorbid migraine in PwMS was associated with geographical location, Sahai-Srivastava et al. noted a higher migraine prevalence in PwMS with a Hispanic ethnicity compared with Whites (82% vs 18%, respectively) [77]. Kipster et al. reported that although migraine status was not significantly associated with migraine-related disability grades or lesion burden, but found that PwMS with migraine followed a more symptomatic course of MS than the no-headache group [70], suggesting that migraine may represent an important determinant of MS progression. Marrie et al. found increased incidence and prevalence of fibromyalgia in PwMS compared with the general population, although this was found to increase over time in both populations [69].

The studies on olfactory dysfunction and ocular comorbidities generally reported increased prevalence in the measured comorbidities, including odor dysfunction, intra-ocular pressure, cataracts, and glaucoma [73, 78, 79]. One study reported that whilst there was no overall risk of cataract in PwMS, the was significantly elevated in patients younger than 50 years of age [74].

### Comorbid autoimmune disease (Supplemental Table 6)

MS shares common risk factors and immunopathologic mechanisms with other autoimmune diseases (AID) and consequently these are over-represented in PwMS and speed its progression [12]. AID reported to coexist with MS include psoriasis, asthma, type 1 diabetes autoimmune thyroiditis, celiac disease, Sjogren’s syndrome, inflammatory bowel disease (IBD), rheumatoid arthritis, systemic lupus erythematosus, and atopic dermatitis. As with MS, psoriasis prevalence appears to show a latitudinal gradient, increasing with greater distance from the equator [80]. Rheumatoid arthritis prevalence is increasing globally, but this is particularly rapid in certain countries in the Americas [81], whereas historically IBD has presented a high prevalence rates in North America and Europe, but now Japan and India, countries with previously low-risk are also seeing an increase in incidence [82].

Studies reporting the epidemiology of comorbid AID in patients with MS (Supplemental Table 6, *N* = 19) comprised a systematic review, as well as several cohort studies and cross-sectional studies that were performed in Europe (*n* = 5), the USA (*n* = 4), Argentina (*n* = 1), Australia (*n* = 1), Canada (*n* = 3), Israel (*n* = 2), and Taiwan (*n* = 2). Although no regional trends were noted, we found that the reported risk and prevalence of AID in general, and for specific conditions, were generally consistent with the literature. The findings in Supplemental Table 6 show significantly increased rates of all AID studied in PwMS, including psoriasis, rheumatoid arthritis, and IBD, although—as with other comorbidities—the reported prevalence rates and measures were heterogenous between studies. Interestingly, Miron et al. reported that psoriasis onset had preceded the MS diagnosis in 78% of PwMS with comorbid psoriasis [83]. Relapse analysis from another study showed that PwMS with comorbid rheumatoid arthritis had a threefold increased risk of MS relapse [14] and that psoriasis, thyroid disease, and type 2 diabetes mellitus comorbidities were all associated with more severe outcomes in PwMS [84]. Although the numbers are relatively small in these studies, these data warrant further investigation.

### Cancer (Supplemental Table 7)

Given that MS and cancer share certain risk factors, such as aberrant T-cell functioning [85], an association between these diseases is feasible, so understanding any potential relationship between the diseases is important. To date, studies investigating the epidemiology of cancer in PwMS have produced conflicting results. Some studies suggest that MS is linked to a reduced cancer rate, but a positive relationship is also found for several types of cancer, such as meningiomas and urinary system cancers [20]. Typically, the prevalence of cancer type varies widely by region, with lung cancer being the most diagnosed cancer for men in Eastern Europe and most of Asia, whereas, in Western Europe and the Americas, it is prostate cancer. This variation is less prominent for women, however, with breast cancer being the most commonly diagnosed cancer across the majority of countries in all regions [86].

Fourteen studies from Europe (*n* = 9), the USA (*n* = 1), Canada (*n* = 2), Iran (*n* = 1), and Taiwan (*n* = 1) investigating the epidemiology of cancer in PwMS (Supplemental Table 7) included matched population studies or MS cohorts compared with expected cancer rates in the general population. Most studies showed no association or a small reduction in cancer risk or prevalence in PwMS [87–91], the exceptions were studies showing an elevated risk of postmenopausal breast cancer in a Swedish population [92] and overall increases in multiple cancer types in a Taiwanese population [93].

Studies investigating DMT use compared PwMS using DMTs with DMT-naïve patients and results suggest that cancer risk may be increased in patients using DMTs [94, 95], although the risk was not consistently observed [89]. Another study showed that cancer risk increased with switching DMTs [96]. Whether MS plays a protective role in developing cancer, or vice versa, remains to be seen, but the molecular mechanisms and possible diverging pathogenesis merit investigation.

### Metabolic disorders, dyslipidemia, diabetes (Supplemental Table 8)

The elevated prevalence of metabolic disorders, such as type 2 diabetes, hyperlipidemia, and insulin resistance may worsen disease in PwMS [11, 97], but the frequency of metabolic comorbidities in PwMS has not yet been fully elucidated. Given that the prevalence of diabetes is expected to increase globally, from 8% in the 2017 to 10% by 2045 (IDF Atlas, 2017), individuals with elevated risk of MS should be carefully monitored for comorbid cardiometabolic disorders, particularly in regions with higher risk of both diseases, such as North America.

We report 11 studies investigating the epidemiology of metabolic disorders in PwMS (Supplemental Table 8) in Europe (*n* = 3), North and South America (*n* = 6), Australia (*n* = 1), and Israel (*n* = 1). The studies comprised cross-sectional studies and both retrospective and prospective cohort studies investigating different aspects of metabolism in PwMS. Findings showed that insulin resistance and fasting glucose concentration were increased in PwMS compared with the control subjects (40.0% vs 21.12% [98] and (17% vs 2% [99], respectively),and high prevalence of dyslipidemia/hyperlipidemia was also reported (prevalence 11.9–30.0%. Contrarily, Marrie et al. [100] observed similar rates of diabetes and hyperlipidemia in PwMS and the general population in a Canadian cohort study [100], and Pinhas-Hamiel et al. reported no difference in prevalence of metabolic syndrome in a population of PwMS in Israel, and moreover, lower rates of obesity and lower body mass index in disabled PwMS [101]. No geographical trends were evident.

Concerning MS prognostic markers, one study found a positive correlation between plasma glucose and both EDSS (*P* = 0.008) the rate of clinical relapse (*P* = 0.001) [102],and another shows that insulin resistance in PwMS was associated with disability as measured using EDSS (*P* = 0.031) and elevated levels of markers of inflammation (*P* = 0.006) and oxidative stress (*P* = 0.029) compared with patients without insulin resistance [98].

### Pulmonary disease and asthma (Supplemental Table 9)

Chronic pulmonary conditions are disruptive to daily living and impact QoL, and these negative influences are compounded in patients with both [103], but their prevalence in PwMS and relationship with MS progression remains unclear. In terms of global prevalence, chronic lung diseases such as chronic obstructive pulmonary disease (COPD) and asthma are rising globally. The greatest burden for COPD is seen in South Western nations in Africa such as Papua New Guinea and Lesotho and countries in the South and East of Asia, such as India, China, and Nepal [104].

Supplemental Table 9 presents the findings from studies investigating the epidemiology of pulmonary disease and asthma in patients with MS (*N* = 11). The studies involved PwMS from Europe (*n* = 3), North America (*n* = 5), Argentina (*n* = 1), Australia (*n* = 1) and Taiwan (*n* = 1). Studies were cohort and cross-sectional in design and reported conflicting findings regarding asthma prevalence in PwMS, with some studies reporting increased prevalence [14, 105] and others reporting no difference [106] or decreases [107], which was further compounded by the lack of control groups in some these studies [106, 108]. However, there was a trend towards an increased prevalence of chronic lung disease [103, 109], although no regional trends were observed.

## Discussion

This review emphasizes a holistic and multidisciplinary approach to viewing and treating MS and its associated comorbid conditions, as autoimmunity and inflammation play interconnected roles in disease pathogenesis. The findings of this systematic review confirm that multi-system comorbidities are frequent features in PwMS and are commonly associated with disease progression.

**Fig. 2:**
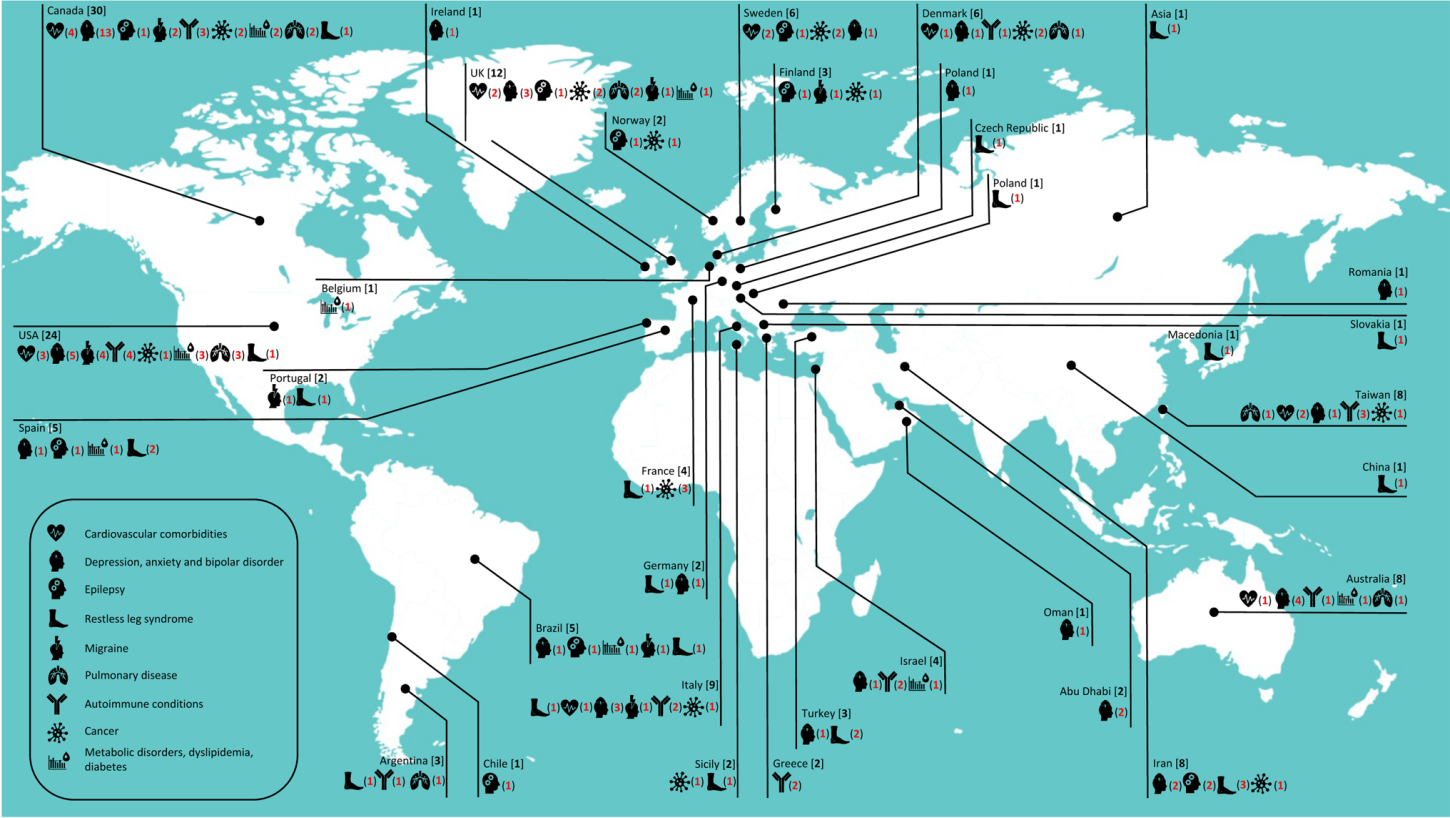
Worldwide distribution of studies on comorbidity in multiple sclerosis

The worldwide distribution of studies on comorbidity in multiple sclerosis is shown in Fig. 2. While multiple studies were conducted in Europe and North America, including many high-powered epidemiologic studies from Canada, Italy, the USA, Sweden, and the UK, establishing region-specific comorbidity trends in PwMS remains a challenge, because most world regions have been inadequately studied. No studies from Africa and few from Asia were found and even within European and North American studies, the data largely originate from a small number of regions within those continents. The latitudinal gradient and lack of funding of studies are likely to be responsible for the under-representation of many regions in MS comorbidity studies. Although the data suggest that Middle Eastern countries such as Abu Dhabi may have lower rates of mood disorders, this warrants further investigation.

### Limitations

Despite the apparent plethora of studies assessing comorbid conditions in PwMS, the findings are heterogenous and often difficult to compare, largely due to methodological differences between studies: different measures, for example absolute prevalence or incidence rates versus risk ratios using statistical inferences (*P* values and confidence intervals); study group sizes were also variable as were time scales (e.g., using defined time points or different follow-up periods), as well as the lack of age-, sex- and ethnicity-specific risk estimates or controls for certain risk factors. Classifications and definitions of the symptoms and comorbidities were also variable, as were the survey methods and diagnostic thresholds used to measure conditions. Other limitations of data were that some findings were obtained via self-report, which may be subject to recall bias instead of objective measures of these conditions, such as physician diagnoses or the results of clinical tests. Despite the limitations and the inter-relatedness of comorbid MS, several interesting findings emerged.

### Multiple comorbidities are highly prevalent in PwMS

Many comorbidities are robustly increased in PwMS, particularly mood disorders, other comorbid autoimmune conditions, and cardiovascular disease, although rates were heterogeneous, so directly comparing results was challenging. Migraine, RLS, and epilepsy were all increased compared with the control population (where provided), with varying rates of prevalence. Metabolic conditions showed mixed results, though tending to be higher in PwMS, and cancer reduced versus the general population.

### Comorbidities are associated with MS progression

Despite their apparently high prevalence, reporting comorbidities in PwMS at the timepoint of diagnosis is rare [110]. The findings from this review suggest that the presentation of common comorbidities should warrant further investigation, particularly with clinical suspicion of MS. The appearance of some comorbidities, such as anemia, epilepsy and bipolar disorder, was reported to precede MS diagnoses [28, 32, 111], and the stage of MS at which comorbidities appear may have a significant bearing on the progression of MS and comorbidities [31]. Physical and psychiatric comorbidities were shown to be associated with reduced health-related QoL. Further several studies suggest that comorbidity is associated with increased mortality.

Comorbidities have an adverse influence on outcome. Different comorbidities were found to be associated with disability and clinical features of disease progression in PwMS [47],RLS was associated with faster MS progression [67], migraine with a more symptomatic course of MS [70], and hypertension with structural brain changes [30] in PwMS. Other studies reported that asthma and rheumatoid arthritis were associated with MS relapse [14], and insulin resistance with both EDSS and inflammatory/oxidative stress markers [98] in PwMS. As with symptoms of MS, comorbidities must be continually monitored as their trajectory can have a significant bearing on MS progression, potentially forecasting exacerbations [31]. The association of socioeconomic status, genetic factors, ethnicity, and health behaviors with disability outcomes and progression are further factors which hinder individual prognostication. Moreover, knowledge about effects of MS on outcomes related to these comorbid conditions is scarce since most studies focused on the influence of comorbidities on the MS trajectory.

### DMT use may interact with the comorbid disease in PwMS

As described, comorbidities in PwMS can impact on DMT adherence and, therefore, and may influence the choice of DMT [3, 112],however, this report suggests that DMT use may also increase the risk of developing comorbidity across several conditions in PwMS. For example, interferon beta and glatiramer acetate uses have been associated with an increased prevalence of cardiovascular risk factors, including elevated diastolic blood pressure and plasma glucose, in addition to altered lipid profiles [29],other studies have suggested that DMT use may increase cancer risk in PwMS [94–96]. Therefore, improved understanding of the effects of comorbidity on safety, the effectiveness of DMT, and potential interactions between DMT use and comorbidity in PwMS is needed.

## Conclusion

These studies have shown the wide range of medical issues faced by PwMS that contribute to premature morbidity, which must encourage enhanced surveillance with a multidisciplinary approach. The lack of large and standardized epidemiologic studies addressing the limitations set out in this report represents a significant barrier to optimal management of PwMS. Comorbid conditions impact the QoL of PwMS and aggressively treating them may hinder MS progression. Therefore, understanding the risk factors for developing such diseases and how these might affect MS progression is key to individualized treatment that could improve outcomes for PwMS.

## Electronic supplementary material

Below is the link to the electronic supplementary material.

Supplementary file1 (DOCX 291 kb)

## Data Availability

All data are included in the manuscript and supplements.
