# Supplementary material for: A global view of comorbidity in multiple sclerosis: a systematic review with a focus on regional differences, methodology, and clinical implications
Source: J Neurol. 2020 Jul 27;268(11):4066–77. doi: 10.1007/s00415-020-10107-y (PMC8505322; doi:10.1007/s00415-020-10107-y)
Supplement: Supplementary file 1 — Supplementary file1 (DOCX 291 kb) [file 415_2020_10107_MOESM1_ESM.docx]

## **Supplemental Tables for comorbidities in MS review manuscript: Global epidemiology of MS comorbidities**

Contents

[Tables for comorbidities in MS review manuscript: Global epidemiology of MS comorbidities 1](#_Toc30700297)

[Table 1: Cardiovascular comorbidities 1](#_Toc30700298)

[Table 2: Psychiatric/ Neurological comorbidities: Depression, anxiety and bipolar disorder 4](#_Toc30700299)

[Table 3: Psychiatric/ Neurological comorbidities: Epilepsy 11](#_Toc30700300)

[Table 4: Restless leg syndrome 14](#_Toc30700301)

[Table 5: Migraine 15](#_Toc30700302)

[Table 6: Autoimmune conditions 17](#_Toc30700303)

[Table 7: Cancer 21](#_Toc30700304)

[Table 8: Metabolic disorders, dyslipidemia, diabetes 23](#_Toc30700305)

[Table 9: Pulmonary disease 26](#_Toc30700306)

[Abbreviations: 28](#_Toc30700307)

[References 28](#_Toc30700308)

### Table 1: Cardiovascular comorbidities

| Reference | Study type | Region | Measure | Outcome |
| --- | --- | --- | --- | --- |
| Roshanisefat et al, 2014  [1] | Population-based cohort study with matched controls  7667 PwMS, 76,045 control subjects from the general population | Sweden | Incidence of cardiovascular disease (CVD) including venous thromboembolism (VTE), stroke, angina, atrial fibrillation (AF). | Overall incidence rates per 1000 person years for CVD are 11.8 (11.06–12.66) for the MS cohort and 8.8 (8.60–9.05) for the non-MS cohort. The most pronounced CVD association was for VTE: relapsing-remitting 2.16 (1.21-3.87; n = 14), secondary progressive 3.41 (2.45-4.75; n = 52) and primary progressive 3.57 (1.95-6.56; n = 15). MS was associated with a decreased relative risk (RR) for angina pectoris (0.72, 95% CI 0.58–0.88) and AF (0.57, 95% CI 0.43–0.74). There is a significantly increased RR of 1.31 for CVD in PwMS (95% CI 1.22─ 1.41). |
| Jadidi et al, 2013  [2] | Population-based cohort study with matched controls.  8281 PwMS, 76,640 control subjects from the general population | Sweden | Risk of myocardial infarction (MI), stroke and heart failure (HF) and AF/flutter | Among MS patients, the incidence rate ratio (IRR) for MI was 1.85 (95% CI 1.59 ─2.15), for stroke was 1.71 (95% CI 1.46─2.00), for HF was 1.97 (95% CI 1.52─2.56) and for AF/Flutter was 0.63 (95% CI 0.46─0.87), as compared with individuals without MS. |
| Christiansen et al, 2010  [3] | Population-based cohort study with matched controls  13,963 PwMS, 66,407 control subjects from the general population | Denmark | Risk of MI, stroke and HF and AF in PwMS | During the first year of follow-up, the risk of MI was 0.2% among patients with MS (adjusted IRR = 1.84, 95% CI 1.28–2.65, compared with population cohort members), whereas the 1-year risk of overall stroke was 0.3% (adjusted IRR = 1.96, 95% CI 1.42–2.71). IRRs were 1.92 (95% CI 1.27–2.90) for heart failure and 0.77 (95% CI 0.42–1.39) for AF/flutter. During the subsequent 2–30 years of follow-up, IRRs remained elevated for overall stroke (1.23, 95% CI 1.10–1.38) and heart failure (1.53, 95% CI 1.37–1.71 but decreased for acute MI (1.10, 95% CI 0.97–1.24). In this Danish cohort, the risk of CVD among MS patients was low, but greater than that in the general population, particularly in the short term. |
| Koudriavtseva et al, 2015  [4] | Retrospective, cross-sectional, case-control study with 187 consecutive PwMS (51 males, mean age (±SD) 44.5 ± 10.7 years) and 200 controls subjects | Italy | Prevalence of anemia relative to controls; relationships between patients’ characteristics and anemia; effect of anemia on the risk of developing MS | There was a significant difference in the prevalence of anemia between MS patients and controls (35 (18.7%) and 19 (9.5%), respectively, p = 0.009). The occurrence of anemia increased more than twice the risk of developing MS (odds ratio (OR): 2.19, 95% CI 1.19–4.0). |
| Peeters et al, 2014  [5] | Population-based matched-cohort study  in 5566 PwMS and 33,370 control patients | UK | Risk of VTE associated with MS and VTE within the MS cohort. Time-dependent adjustments were made for age, comorbidity, and medication use. | 2.6-fold increased risk of VTE was observed in PwMS compared with control cohort (adjusted hazard ratio [AHR] 2.56, 95% CI 2.06–3.20). Within the MS cohort, a recent record of spasticity (AHR 2.59, 95% CI 1.72–3.91) or disability (AHR 2.04, 95% CI 1.26–3.31) was associated with VTE. Furthermore, the risk of VTE was higher in patients who had recently been exposed to high-dose glucocorticoids (AHR 2.58, 95% CI 1.36–4.88). No signiﬁcant relationship was observed between the risk of VTE and MS disease duration (from the ﬁrst MS diagnosis). |
| Jick et al, 2015  [6] | Cohort study in 1713 PwMS.  No control group | UK | Assessment of lifetime incidence of comorbidity in PwMS | Following MS diagnosis, frequent comorbidities were infections (80 %), and depression (46 %). Incidence of chronic comorbidities recorded at any time in the database were: Chronic obstructive pulmonary disease (COPD) and asthma (20.0%) depression (45.8%) diabetes (5.4%) hypertension (14.8%) heart disease (3.9%) cancer (6.4%)  No control group was present for comparison. |
| Sternberg et al, 2014  [7] | Retrospective chart review of PwMS taking DMTs (188) vs DMT naïve PwMS (110) | USA | Assessment of CV risk factors associated with DMT use in PwMS | DMT use was associated with higher diastolic BP readings (p=0.03), as well as higher plasma glucose (p=0.02) , lower HDL-C plasma levels (p=0.01) in PwMS taking DMTs vs DMT-naïve PwMS. In addition, there was an association between CV risk factors and the type of DMTs: the use of IFN-b (12.5 vs. 18.6%, p = 0.05), GA (5.7 vs. 18.6%, p = 0.02), and NTZ (3.7 vs.  18.6%, p = 0.01) was associated with lower percentages of patients who had HDL-C plasma levels below 40 mg/dL, compared to DMT-naive patients. In DMT-naïve patients, the use of CV and related drugs was associated with higher Extended Disability Status Scale (EDSS) and higher MS Severity Scale (MSSS) (P<0.05). |
| Sternberg et al, 2013  [8] | Retrospective chart review of 206 PwMS s vs 142 control patients with meningiomas and acoustic neuromas, non-inflammatory, non-autoimmune diseases of the brain | USA | Comparison of CV risk factors in MS and non-MS patients | MS patients had significantly higher total plasma cholesterol (p = )0.01, and plasma high density lipoprotein (p <0.001), but lower plasma glucose, (p <0.001), and systolic BP, (p = 0.001), than non-MS patients. In addition, MS patients had lower erythrocyte sedimentation rate and serum vitamin B12, but higher serum folic acid and vitamin D3 than non-MS patients (p≤0.05). A positive correlation was observed between plasma glucose and the extended disability status scale (EDSS), (p= 0.008), and between plasma glucose and the rate of clinical relapse, (p=0.001) |
| Kappus et al, 2016  [9] | In a prospective study, 326 patients with relapsing–remitting MS and 163 patients with progressive MS, 61 patients with clinically isolated syndrome (CIS) and 175 healthy controls (HCs) | USA | Evaluation of the association between individual or multiple CV risks and MRI outcomes was examined adjusting for age, sex, race, disease duration and treatment status. | PwMS showed increased frequency of smoking (51.7% vs 36.5%, p=0.001) and hypertension (33.9% vs 24.7%, p=0.035) compared with HCs. In total, 49.9% of patients with MS and 36% of HCs showed ≥2 CV risks (p=0.003), while the frequency of ≥3 CV risks was 18.8% in the MS group and 8.6% in the HCs group (p=0.002). In patients with MS, hypertension and heart disease were associated with decreased grey matter (GM) and cortical volumes (p<0.05), while overweight/obesity was associated with increased T1-LV (p<0.39) and smoking with decreased whole brain volume (p=0.049). Increased lateral ventricle volume was associated with heart disease (p=0.03) in CIS. |
| Marrie et al, 2019  [10] | Population-based cohort study in 14,565 PwMS 72,825 matched control subjects | Canada | Risk of acute myocardial infarction (AMI) | The crude incidence of AMI per 100,000 population was 146.2 (95% CI 129.0–163.5) in the MS population and 128.8 (95% CI 121.8–135.8) in the matched population. Risk of AMI was 60% higher in PwMS as compared to an age-, sex-, and geographically matched population without MS (hazard ratio [HR] 1.63; 95% CI 1.43–1.87). |
| Marrie et al, 2013  [11] | Population-based matched-cohort study in 2.366 PwMS and 11,786 control subjects from the general population | Canada | Prevalence and incidence of ischemic heart disease (IHD) in MS vs general population, also stratified by age | The age-standardized prevalence of IHD was 6.77% (95% CI 5.48–8.07) in PwMS  and 6.11% (95% CI 5.56–6.66) in the general population. The prevalence of IHD was higher in the MS patients than the general population among persons aged 20-44 years (prevalence ratio [PR] 1.87, 95% CI 1.65─2.12) and aged 45-59 years (PR 1.21, 95% CI 1.08─1.35). The incidence of IHD was also higher in the MS patients (IRR 1.24; 95% CI: 0.97-1.59). More than 5% of the MS patients has IHD. The prevalence of IHD was higher than expected in persons aged <60 years |
| Kowalec et al, 2017  [12] | 2-year prospective multicenter cohort study in 885 PwMS  No control group | Canada | Cross-sectional assessment of comorbidities and relapses. Comorbidities were recorded using questionnaires, and relapses were captured from medical records at each visit. | Anxiety (40.2%), depression (21.1%), hypertension (17.7%), migraine (18.1%), and hyperlipidemia (11.9%) were the most prevalent comorbidities.  Migraine and hyperlipidemia were associated with increased relapse rate (adjusted rate  ratio 1.38; 95% CI 1.01–1.89 and 1.67, 95% CI 1.07–2.61, respectively)  No control group was present for comparison |
| Marrie et al, 2016  [13] | Population-based study of 23,382 PwMS and 116,638 matches from the general population | Canada | Prevalence of comorbidity in PwMS from administrative health data from 4 Canadian provinces, including British Columbia, Manitoba, Quebec, and Nova Scotia. | Crude prevalence of comorbidity in MS at diagnosis compared to matched controls. Hypertension (15.2% vs 12.9%, RR 1.17 (95% CI 1.13─1.21, p<,0.0001); diabetes (5.69% vs 4.86%, RR 1.17 (95% CI 1.10─1.24, p<0.0001); IHD (6.50% vs 5.01, RR 1.30 (95% CI 1.23─1.37, p<,0.0001); fibromyalgia (1.31% vs 0.46%, RR 2.87, 95% CI 2.49 ─3.30, p<0.0001); IBD (0.56% vs 0.30%, RR 1.68, 95% CI 1.38─2.04 p<0.0001). Chronic lung disease (12.1% vs 9.14%, RR 1.34, 95% CI 1.29─1.39); epilepsy (1.93 vs 0.89%, RR 2.18, 95% CI 1.95, 2.43, p<0.0001; depression (19.1% vs 9.38%, RR 2.04, 95% CI 1.97─2.10, p< 0.0001); Anxiety (11.1% vs 6.89%, RR 1.61, 95 CI% 1.54─1.68, p<0.0001; bipolar disorder (3.15% vs 1.69%, RR 1.86, 95% CI 1.71─2.02 p<0.0001; schizophrenia (1.07% vs 0.81%, RR 1.32, 95% CI 1.15─1.52, p<0.0001) in PwMS vs control population, respectively. |
| Tettey et al, 2016  [14] | Prospective cohort of 198 PwMS | Australia | Evaluation of specific doctor-diagnosed comorbidities in PwMS versus the 207 general population prevalence | The age-standardized prevalence of hypertension (21.21% vs 9.43%), dyslipidemia (14.65% vs 5.72%), asthma (18.69% vs9.93%), psoriasis (7.58% vs 2.29%), eczema (13.13% vs 0.92%) and anemia (12.63% vs 1.83%) were significantly higher in the MS cohort compared to that in the general Australian population (p≤0.001.  For relapse analyses, rheumatoid arthritis and anemia were associated with more than threefold (HR 3.70, 95% CI 1.80─7.58, p=0.001) and twofold (HR 2.04, 95% CI 1.11─3.74, p = 0.022) increased risk of subsequent relapse respectively. The prevalence of some comorbidities was higher in MS patients and associated with greater disability and relapse risk. |
| Tseng et al, 2014  [15] | Nationwide controlled cohort study in 1174 PwMS and 4696 control patients | Taiwan | Assessment of insurance registry data (1174 MS patients) vs 4696 from general population | The overall incidence rates for ischemic stroke in the study and control cohorts were 6.45 and 1.60 per 1000 person-years, respectively MS cohort had an increased risk of stroke (adjusted HR = 12.1 (95% CI 3.47 −42.1 for 1 year; adjusted HR = 4.69 for 2-5 years, 95% CI 2.51 ─8.76) compared with the control cohort within 5 years of follow-up. The 1-year and 5-year incidence rates of stroke were 9.96 and 8.12 per 1000 person-years, respectively, in MS patients and 0.90 and 1.48 per 1000 person-years, respectively, in the controls. In the population aged ≤40, MS was associated with a significantly increased risk of stroke (HR 12.7, 95% CI 3.44-46.7). |
| Kang et al, 2010  [16] | Cohort study in 898 PwMS and 4490 control subjects | Taiwan | Evaluation of 30 comorbid medical conditions from insurance claims | PwMS were more likely to have systemic lupus erythematosus (OR = 26.9, 95% CI 10.3−70.3), depression (OR = 6.9, 95% CI 5.3─8.9), peripheral vascular disorders (OR = 6.6, 95% CI 4.0−11.0), deficiency anemias (OR = 4.9, 95% CI 2.8−8.7), rheumatoid arthritis (OR = 4.8, 95% CI 2.9−8.1) and fluid and electrolyte disorders (OR = 4.8, 95% CI 2.8−8.3) than the matched controls. Patients with MS had higher risk of multiple medical comorbidities compared to a matched control group in an ethnic Chinese population. |
|  |  |  |  |  |

Abbreviations listed in appendix

### Table 2: Psychiatric/ Neurological comorbidities: Depression, anxiety, bipolar disorder and obsessive compulsive disorder

| Reference | Study type | Region | Measure | Outcome |
| --- | --- | --- | --- | --- |
| Ben Ari Shevil, 2014 [17] | Observational, prospective study in 200 PwMS  No control group. | Sweden | Assessment of functioning, disability and utilization of health-related services in PwMS | 17.8% had signs of depression  No control group was present for comparison. |
| O’Connell et al. 2017  [18] | Prospective, multicenter, population-based, observational study in 292 PwMS.  No control group | Republic of Ireland | Evaluation of the prevalence of depression in PwMS using HADS over a 12-month period | Depressive symptoms were reported in 34% of PwMS with no prior history of depression  No control group was present for comparison. |
| Solaro et al, 2016  [19] | Multicenter MS population study  1,011 PwMS.  No control group. | Italy | Investigation of the prevalence of depressive symptoms in a multicenter MS population using the BDI II and identification of possible correlations between  the BDI II score and demographic and clinical variables. | 343 (33.9%) scored greater than 14. For patients with BDI>14 multivariate analysis showed a significant difference between EDSS and disease course (p≤.0.001).  No control group was present for comparison. |
| Mattioli et al, 2011  [20] | Cross-sectional case-matched study in 255 PwMS and 166 control subjects | Italy | Evaluation of depressive symptoms, its predicting factors and relation with cognitive impairment, fatigue and disability in a sample of PwMS.  Patients with BDI-FS ≥ 4 were further investigated for the presence of neuropsychological impairment. | 25.5% of MS patients were depressed compared with 15.6% of general population (p=0.017). EDSS score was the only predicting factor of depression (3.5 = threshold EDSS score for depressive symptoms) in patients. The RR for depression in PwMS was (1.248, 95% CI 1.09−1.42) indicating for any point in the EDSS an increasing risk of depression of 25%, whereas neuropsychological impairment was not correlated with BDI-FS (p>0.24) and fatigue was found to be significantly correlated with attention, executive function and memory test scores, as well as with BDI-FS score in patients (p≤0.05). |
| Rossi et al, 2017  [21] | Cross sectional study of 405 PwMS. No control group | Italy | Assessment of anxiety prevalence and association with neuroinflammation using BDI-II, STAI-Y.  Cytokines levels and MRI (detecting neuroinflammation) | Clinically significant state anxiety prevalence was 33.5%, whereas the trait anxiety prevalence was 28.1%. A significant reduction of STAI-state and BDI-II scores was recorded, along with the subsiding of neuroinflammation (p<0.001. Interleukin-2CSF levels were found to correlate with STAI-state, while tumor necrosis factor-a and interleukin-1b correlated with BDI-II (p<0.001.  No control group was present for comparison. |
| Jick et al, 2015  [6] | Cohort study in 1713 PwMS.  No control group | UK | Assessment of lifetime incidence of comorbidity in PwMS | Following MS diagnosis, frequent comorbidities were infections (80 %), and depression (46 %). Incidence of chronic comorbidities recorded at any time in the database were: Chronic obstructive pulmonary disease (COPD) and asthma (20.0%) depression (45.8%) diabetes (5.4%) hypertension (14.8%) heart disease (3.9%) cancer (6.4%)  No control group was present for comparison. |
| Garfield et al. 2012  [22] | Cross sectional study, 157 PwMS  No control group | UK | Identification of factors associated with the presence of anxiety in PwMS using HADS, GHQ-12, and PSS | Of the 157 participants who took part, 89 (57%) were clinically anxious. Participants who were anxious had a lower level of self-efficacy (p < 0.001), higher level of  disability (p < 0.001), higher level of depression (p < 0.001) and higher level of stress (p < 0.001). Depression (χ(2) = 5.05, OR = 1.32, p< 0.05) was the only factor that significantly predicted whether someone was anxious or not, accounting for 46% of the variance  No control group was present for comparison. |
| Sicras-Mainar et al, 2017  [23] | Retrospective cohort study using electronic medical records with 222 PwMS.  No control group | Spain | Estimation of the presence  of comorbid conditions and metabolic syndrome (MetS) in a sample of adult patients with MS. | Depression (32.4%), dyslipidemia (31.1%), hypertension (23.0%) and obesity (22.5%) were the most common comorbidities. Overall MetS prevalence was 31.1% (95% CI 25.0−37.2%). Patients with an EDSS ≥ 4.0 showed a significantly higher number of comorbidities (OR=2.2, 95% CI: 1.7−3.0; p<0.001).MS patients had a high prevalence of MetS.  No control group was present for comparison. |
| Schmidt et al, 2019  [24] | Single center cross-sectional study in 260 PwMS  No control group | Germany | Assessment of prevalence of depression and fatigue in PwMS and their association with QoL, used MusiQoL, CES-D, and FSMC | In this group pf PwMS 35.8% had depression and 56.9% moderate to severe fatigue. Using linear regression, depression, fatigue, family status, physical activity, and occupation were associated with QoL (p<0.05).  No control group was present for comparison. |
| Kale et al, 2009  [25] | Small prospective study in 57 PwMS  No control group | Turkey | Estimation of depression prevalence and association with fatigue and outcomes FSS, EDSS, and BDI. | Clinically relevant depression (BDI>18) was present in 28 (49%). Patients were divided into three groups: FSS score >5 (n=10, 32%) were evaluated to present with fatigue symptoms, patients with borderline fatigue (n=29, 50%) had an FSS score between 4 and 5 and patients with no fatigue (n=18, 18%) had an FSS<4. MS patients with fatigue showed significantly higher BDI scores when compared to patients without fatigue (p=0.0002).  No control group was present for comparison. |
| Maier et al, 2016  [26] | 351 PwMS  No control group | Romania | Assessment of the  prevalence of depression in PwMS and the physical and psycho-socioeconomic  factors that influence its frequency and severity. Used BDI-II and EDSS | We found a strong association between depression and the severity of MS as indicated by EDSS. Of the eight functional systems of the EDSS, the pyramidal and sensitive scores had the strongest association with depression level. In a logistic regression analysis, the OR for clinically signiﬁcant depression for pyramidal symptoms was 1.39 (95 % CI 1.04–1.87, p = 0.02) and 1.35 for sensitive symptoms (95 % CI 1.03–1.77, p = 0.03).  No control group was present for comparison. |
| Hoang et al, 2015  [27] | Nationwide registry cohort of 5084 PwMS and matched with a control population of 24,771 | Denmark | Estimation of the risk of depression and anxiety and the use of tricyclic antidepressant and  selective serotonin reuptake inhibitors (SSRI), in the pre- and post-diagnostic  MS compared to the background population | In the pre-diagnostic period, the OR for having a diagnosis of depression and anxiety is 1.4  (95% CI 1.05–1.88) and the OR of redemption prescriptions of TCAs is 1.90 (95% CI 1.54–2.34) and OR is 1.34 (95% CI 1.20–1.51) for SSRI. In the post-diagnostic period, the OR is 1.23  (95% CI 0.92–1.64) for depression and anxiety diagnosis. The OR is 6.70 (95% CI 5.81–7.72) for TCA and OR is 2.46 (95% CI 2.25–2.69) for SSRI. |
| Persson et al, 2019  [28] | Population-based cohort study with matched controls  8695 PwMS, 86,934 controls in DOD database; 6932 PwMS, 68,526 controls in GOLD database | USA and UK | Assessment of the prevalence of depression in PwMS versus the general population | The prevalence of depression in MS patients (~ 21%) was higher than in non-MS patients (~16%))( p < 0.01) |
| Alschuler et al, 2012  [29] | Cross sectional community-based survey of 117 PwMS | USA | Evaluation of the association of depression with pain treatment utilization in  an MS population. PHQ-9, NRS | 25.9% scored PHQ-9 ≥ 10 and 11.1% endorsing enough items to meet DSM-IV criteria for a major depressive episode. Depression was not associated with higher pain  treatment utilization. |
| Newland et al, 2015  [30] | Cross-sectional study of 339 PwMS compared with normal population | USA | Comparison of the % of health conditions between  PwMS and U.S. national norms | Rates of depression (55% vs 3%, p<0.0001), cancer (15% vs 9%, P=0.01), migraine (37% vs 17%, b) and arthritis (32% vs 25%, p=0.003) are higher in MS than the US norm |
| Jun-O’Connell et al, 2017  [31] | Cross-sectional study of 152 PwMS  No control group | USA | Evaluation of the prevalence of BD among patients with MS using psychiatric diagnostic interviews (MDQ), and evaluates QoL | Higher prevalence of BD in patients with MS compared to the general population (16 PwMS screened positive on the MDQ (10%), and 10 PwMS (6.5%) scored positive for BD via SCID (p<0.001). Bipolar disorder type 1 was more prevalent than type 2 in the study group (60% versus 30%, p <0.001). The presence of BD was associated with significantly reduced MSQOL-54 physical and mental composites (p=0.003, and p<0.001 respectively).  No control group was present for comparison. |
| Burns et al, 2013  [32] | Secondary analysis of 121 patients with MS who were followed for 48 weeks during a randomized controlled trial. No control group | USA | Determination of whether pseudo- and confirmed MS exacerbations were preceded by or concurrent with increased anxiety or depressive symptoms | The prevalence of generalized anxiety disorder was 22/121 (18%) and major depressive disorder was 16/121 (13%). A standard deviation increase in anxiety symptoms relative to baseline predicted subsequent onset of pseudo-exacerbations, OR=1.54, p= 0.02, while increased somatic depressive symptoms predicted confirmed exacerbations [OR]=1.59, p=0.01.  No control group was present for comparison. |
| Edwards et al, 2016  [33] | Observational longitudinal survey study. 489 PwMS  No control group | Canada | Investigation of whether baseline levels of pain, fatigue, sleep disturbance and physical activity measured at the initial assessment predicted the development of or improvement of  depression. EDSS and PHQ-9 with a cut-off of 10 for probable major depression; GLTEQ; PROMIS | The baseline prevalence of probable major depression was 26% (N= 128). Fatigue severity (OR, 1.19, 95% CI 1.12−1.26, P<0.0001) and sleep disturbance (OR 1.06, 95% CI 1.02–1.10, P=0.001) predicted probable major depression 3.5 years later among those not depressed at the initial assessment  No control group was present for comparison. |
| Berzins et al, 2017 [34] | Prospective cohort study of 192 PwMS  No control group | Canada | Assessment of depressive symptoms in real-time using PHQ | 2-week incidence of depression for females was 0.019 (95% CI 0.013-0.029) and for  males was 0.044 (95% CI 0.026-0.074). Strongest predictor of depression incidence risk included fatigue impact, low mobility, resiliency, self-esteem, self-efficacy, and coping style  No control group was present for comparison. |
| Gill et al, 2019  [35] | Retrospective chart review of 128 MS PwMS  No control group | Canada | Evaluation of the impact of anxiety and depression on outcomes for PwMS using HADS-A and HADS-D | 38% of participants met the cut-off for depression and 57% met the cut-off for anxiety (HADS-A/HADS-D =8). Higher depressive symptoms were associated with higher fatigue and physical disability and with lower employment and processing speed, whereas higher anxiety symptoms were related to lower physical disability and remaining employed, with fewer restrictions (p<0.05). HADS-A (mean=8.41 [SD=3.98]) and HADS-D (mean=6.23 [SD=3.85]) scores were strongly positively correlated with each other (r=0.52, p<0.05. Depressive symptoms were associated with higher fatigue and physical disability and with lower employment and processing speed (p<0.05). HADS-A was negatively associated with EDSS) and positively associated with vocation (p<0.05)  No control group was present for comparison. |
| Théaudin et al, 2015  [36] | Retrospective chart review in 711 PwMS  No control group | Canada | Prevalence of psychiatric sequalae in PwMS and effects of gender. HADS-A and  HADS-D | 37.8% of PwMS were depressed, 47.7% were anxious and 29.5% were both anxious and depressed, more females were anxious than males (p<0.001).  No control group was present for comparison. |
| Koch et al, 2014  [37] | Longitudinal cohort study. 1376 PwMS at baseline, 984 at one year of follow-up and 967, 457, and 258 at two, three, and four years of follow-up.  No control group | Canada | Long term prognosis of depression over four years of follow-up using CES-D scale | Between 22% and 29% of patients were classified as depressed during the study. Depression at baseline was the strongest predictor of depression at follow-up with Pearson correlation  Coefficients 0.62 −0.68 (p< 0.0001 for all follow-up periods).  No control group was present for comparison |
| Viner et al, 2014  [38] | Community-based cohort of 630 PwMS  No control group | Canada | Point prevalence of depression using PHQ-9 | The prevalence of depression was 26.0% (95% CI 18.9%−33.0%). Depressed subjects had lower quality of life; an increased frequency of suicidal ideation; and more often reported a negative disease course, high stress, low social support and stigmatization. |
| Pham et al, 2018  [39] | Cross sectional study, 244 PwMS  No control group | Canada | Prevalence of anxiety and depression in PwMS and their association using HADS-A and PHQ-9 | The prevalence of anxiety was 28.7%, with a mean score of 6.2 (SD: 4.0, median:6.0, range 0–1 9). The prevalence of depression was 18.5% (mean: 6.5, SD: 5.7, median: 5, range 0–27). Of all patients that reported having anxiety, 53.6% did not have depression. Depression was found to be associated with higher odds of anxiety (OR: 7.31 95% CI) 3.29–16.26)  No control group was present for comparison |
| Marrie et al, 2017  [40] | Population study with 1922 persons with incident MS from 1989 to 2012, and 11,392 age, sex and geographically matched controls from the general population. | Canada | Estimation of the annual prevalence of depression and anxiety disorder using population-based administrative (health claims) data from Manitoba | MS patients had an elevated annual prevalence ratio of depression (1.77; 95% CI 1.64−1.91), and anxiety disorders (1.46; 95% CI 1.35−1.58). |
| Morrow et al, 2016  [41] | Retrospective chart review of 151 PwMS  No control group | Canada | Prevalence of anxiety and depression in PwMS and their interaction, using HADS-A and HADS-D, EDSS and FSS | Prevalence of anxiety in the affected range was 53.6% and depressive symptoms in the affected range was 34.4% and these scores correlated significantly with each other (r=0.574, p,0.001) and with the FSS (r=0.539, p<0.001). HADS-D correlated significantly with EDSS (r=0.259, p=0.001).  No control group was present for comparison |
| Kowalec et al, 2017  [12] | 2-year prospective multicenter cohort study in 885 PwMS  No control group | Canada | Cross-sectional assessment of comorbidities and relapses. Comorbidities were recorded using questionnaires, and relapses were captured from medical records at each visit. | Anxiety (40.2%), depression (21.1%), hypertension (17.7%), migraine (18.1%), and hyperlipidemia (11.9%) were the most prevalent comorbidities.  Migraine and hyperlipidemia were associated with increased relapse rate (adjusted rate  ratio 1.38; 95% CI 1.01–1.89 and 1.67, 95% CI 1.07–2.61, respectively)  No control group was present for comparison |
| Marrie et al, 2016  [13] | Population-based study of 23,382 PwMS and 116,638 matches from the general population | Canada | Prevalence of comorbidity in PwMS from administrative health data from 4 Canadian provinces, including British Columbia, Manitoba, Quebec, and Nova Scotia. | Crude prevalence of comorbidity in MS at diagnosis compared to matched controls. Hypertension (15.2% vs 12.9%, RR 1.17 (95% CI 1.13─1.21, p<,0.0001); diabetes (5.69% vs 4.86%, RR 1.17 (95% CI 1.10─1.24, p<0.0001); IHD (6.50% vs 5.01, RR 1.30 (95% CI 1.23─1.37, p<,0.0001); fibromyalgia (1.31% vs 0.46%, RR 2.87, 95% CI 2.49 ─3.30, p<0.0001); IBD (0.56% vs 0.30%, RR 1.68, 95% CI 1.38─2.04 p<0.0001). Chronic lung disease (12.1% vs 9.14%, RR 1.34, 95% CI 1.29─1.39); epilepsy (1.93 vs 0.89%, RR 2.18, 95% CI 1.95, 2.43, p<0.0001; depression (19.1% vs 9.38%, RR 2.04, 95% CI 1.97─2.10, p< 0.0001); Anxiety (11.1% vs 6.89%, RR 1.61, 95 CI% 1.54─1.68, p<0.0001; bipolar disorder (3.15% vs 1.69%, RR 1.86, 95% CI 1.71─2.02 p<0.0001; schizophrenia (1.07% vs 0.81%, RR 1.32, 95% CI 1.15─1.52, p<0.0001) in PwMS vs control population, respectively. |
| McKay et al, 2015  [42] | Prospective multisite longitudinal study in 949 PwMS  No control group | Canada | Evaluation of the associations between cigarette smoking, alcohol use, and depression and anxiety in MS, using HADS-A and HADS-D | Over the entire study, 53.8% of participants met the HADS-Anxiety criterion and 35.1% met the HADS-Depression criterion at some point (baseline, year one, or year two). Alcohol dependence was associated with depression and anxiety (OR=1.84, 95% CI 1.3−2.58 and OR=1.53, 95% CI 1.05–2.23, respectively) as was smoking (OR=1.37,95% CI 1.04–1.78 and OR= 1.29; 95% CI 1.02–1.63, respectively).  No control group was present for comparison |
| McKay et al, 2018  [43] | Long-term retrospective cohort, with 2312 PwMS  No control group | Canada | Prevalence of psychiatric disorders study using inked MS-speciﬁc clinical and population-based health administrative databases | Mood or anxiety disorder 827 (35.8%); Depression 855 (37.0%); Anxiety 511 (22.1%);  Bipolar disorder 119 (5.1%). The presence of a mood or anxiety disorder was associated with a higher EDSS score (p=0.0002).  No control group was present for comparison |
| De Cerqueira et al, 2015  [44] | Cross-sectional study in 60 PwMS  No control group | Brazil | Evaluation of the frequency of psychiatric disorders BDI, DAI, and EDSS | 36.6% had depression (18.5% showing a current depressive episode, and 18%  with at least one episode of depression), 13.3% had bipolar disorder (BD),  16.7% had GAD and 3.3% had panic disorder (PD), suicide risk was detected in 16.6%.)  No control group was present for comparison. |
| Wood et al, 2013  [45] | Population-based longitudinal cohort with 198 PwMS followed 6-monthly for 2.5 years. No control group | Australia | Estimation of the prevalence of anxiety, depression and fatigue in a representative sample of PwMS, and in subgroups defined by age, sex and disease duration, at cohort entry and over time and how these factors clustered together. HADS with anxiety (cut-point >7) and depression (>7), and FSS | At cohort entry, prevalence of anxiety was 44.5% (95% CI 37−51), depression 18.5% (95% CI 12.6−23.4), and fatigue 53.7% (95% CI 47−61). Fatigue was more common in males than females (RR 1.29, p=0.01), with attenuation of the effect after adjustment for Expanded Disability Status Scale (adjusted RR 1.18, p=0.13). Prevalence of anxiety (but not depression or fatigue) decreased by 8.1% per year of cohort observation (RR 0.92, 95% CI 0.86–0.98, p=0.009), with the effect more pronounced in women (14.6%, RR 0.85, 95% CI 0.79–0.93, –<0.001) than men (2.6%, RR 1.03, 95% CI 0.90–1.17, p=0.77).  No control group was present for comparison. |
| Taylor et al, 2015  [46] | Cross-sectional study in 2459 PwMS.  No control group. | Australia | Investigation of the association between lifestyle risk factors, medication and depression risk, used PHQ-2 | 19.3% of the sample screened positive for depression (PHQ-2 score ≥3). Positive lifestyle factors such as healthy diet, exercise, low alcohol consumption and not smoking, vitamin D, Omega 3 type and fish consumption, meditation and social support were negatively associated with depression in PwMS (p<0.001)  No control group was present for comparison. |
| Knippenberg et al, 2014 [47] | Longitudinal cohort study in 198 PwMS  No control group. | Australia | HADS-A and HADS-D, serum 25(OH)D was measured, and FSS | The prevalence of depression, anxiety and fatigue was 19.1%, 45.2% and 53.7%, respectively. Unadjusted and adjusted de-seasonalized continuous 25(OH)D levels were not associated with depression or fatigue scores (p= 0.18−0.42 and p=0.90, respectively Table 2). However, adjusted categorical 25(OH)D levels higher than 80 nM were inversely associated p=0.015). Higher levels of reported personal sun exposure in the current season were associated with lower depression score.  No control group was present for comparison |
| Simpson et al, 2019  [48] | Longitudinal prospective cohort study in 2224 PwMS, 1441 at 2.5-year review (of whom 1264 completed)  No control group. | Australia | Examination of the relationship of demographic and clinical factors with positive depression-screen  and change in depression over 2.5 years, used PHQ2 and PHQ9 | At 2.5-year review, the prevalence of PHQ-2 positive depression-screen was 14.5%, significantly decreased from that seen at baseline (19.1%, p=0.002). However, the prevalence of PHQ-9 positive depression-screen at 2.5-year review was higher (21.7%).  No control group was present for comparison |
| Kalron et al, 2018  [49] | Cohort study in 122 PwMS.  No control group | Israel | Involvement of mood disorders on falling in PwMS | 38 PwMS (31.1%) were classiﬁed as depressed (mean HADS 11.1, SD=3.4); 52(42.6%) were classiﬁed as anxious (mean HADS 11.1, S.D=3.1) and 56 (45.9%) were neither depressed nor anxious. PwMS categorized in the anxiety/non-depressed subgroup were 6 times less likely  to fall than PwMS without depression or anxiety (OR=0.160, 95% CI 0.040–0.646; p=0.010).  No control group was present for comparison. |
| Askari et al, 2015  [50] | Cross sectional study in 180 PwMS  No control group. | Iran | Evaluation of anxiety in PwMS and associated risk factors, using BDI-II | 48 (26.7%) had BAI score between 0−7, 38 (21.1%) had BAI score between 8−15, 44 (24.4%) had BAI score between 16−25 and 50 (27.8%) had BAI between 26-63. There was significant positive correlation between EDSS and BDI and BAI scores (P<0.001).  No control group was present for comparison. |
| Seyed Saadat, 2014  [51] | Cross-sectional study in 160 PwMS  No control group | Iran | Determination of the prevalence and associated factors of depression, and status of antidepressant use, with BDI-II | The prevalence of depression and severe depression was 59.4 and 18.1 %, respectively, only 21.1 % of patients were on antidepressant Treatment. Fatigue (P<0.0001, OR = 5.98, 95% CI = 2.9–12.3) and older age (p = 0.027, OR =2.24, 95% CI 1.09–4.6) were associated with depression in PwMS.  No control group was present for comparison. |
| Schiess et al, 2019  [52] | Retrospective medical record review of 416 PwMS versus the general population of Abu Dhabi | Abu Dhabi | Records were assessed for documentation of depression in their doctors’ clinical notes or had depression coded as an ICD-9 number | Of the total cohort, 45 (10.8%) had depression. Of the total female patients in the study, 15.3% had depression compared with only 2.7% of the males. The anxiety rate in the cohort (4.8%) was lower than that in the general Abu Dhabi population (18.7%). |
| Alsaadi et al, 2015  [53] | Prospective study of 80 PwMS and 80 control subjects | Abu Dhabi | Rates, patterns, and risk factor of depression and anxiety using PHQ-9, GAD | 17.5% and 20.0% of 80 patients seen in MS clinic had scores consistent with major depression and anxiety disorders, compared with 16.2% and 1.05%, respectively, in control subjects |
| Al-Asmi et al, 2015  [54] | Cross-sectional study in 57 PwMS and 53 healthy control subjects | Oman | Investigation of the prevalence of anxiety, depression and related disabilities among PwMS using HADS-A, HADS-D, and EDSS | The estimated prevalence of anxiety and depression in PwMS (50.8% and 35.1%, respectively) were significantly (p≤0.05) higher than that in the control group (26.4% and 18.9%, respectively). The MS group scored significantly higher than controls on HADS measurements of depression and anxiety (p=0.05 and p=0.01, respectively). |
| Kang et al, 2010  [16] | Cohort study in 898 PwMS and 4490 control subjects | Taiwan | Evaluation of 30 comorbid medical conditions from insurance claims | PwMS were more likely to have systemic lupus erythematosus (OR = 26.9, 95% CI 10.3−70.3), depression (OR = 6.9, 95% CI 5.3─8.9), peripheral vascular disorders (OR = 6.6, 95% CI 4.0−11.0), deficiency anemias (OR = 4.9, 95% CI 2.8−8.7), rheumatoid arthritis (OR = 4.8, 95% CI 2.9−8.1) and fluid and electrolyte disorders (OR = 4.8, 95% CI 2.8−8.3) than the matched controls. Patients with MS had higher risk of multiple medical comorbidities compared to a matched control group in an ethnic Chinese population. |

### Table 3: Psychiatric/ Neurological comorbidities: Epilepsy

| Reference | Study type | Region | Measure | Outcome |
| --- | --- | --- | --- | --- |
| Gasparini et al, 2017  [55] | Meta-analysis and systematic review  2845 PwMS (comparing those with and without epilepsy) without epilepsy as a control group | NA | Epilepsy prevalence in MS and risk factors in PwMS | 217/2845 had epilepsy (7.6%). MS patients with epilepsy had a younger age at onset compared to MS patients without seizures (difference in means = -5.42 years, 95% CI -7.19 to -3.66, p < 0.001). Mean EDSS value at inclusion tended to be higher in patients with epilepsy, without reaching statistical significance (difference in means = 0.45, 95% CI -0.01 to 0.91, p = 0.054).  The MS-epilepsy group was compared with the MS without epilepsy as a control group |
| Krökki et al, 2013  [56] | Population based cohort of 491 PwMS diagnosed 1990 and 2010  No control group | Finland | Measurement of the prevalence of one or more neurological condition | One or more neurological comorbid disease was present in 17.1% of patients (n=84). The prevalence of epilepsy in MS patients was 4.7%, which is greater than that in the general population. Migraine was significantly more common in women with a benign MS course when compared to other types of MS (p=0.046). A significant association between peripheral nervous system disorders and primary progressive MS was found (p=0.027). An association between  Stroke prevalence and the duration of MS disease was also detected (p=0.023).  No control group was present for comparison. |
| Burman et al, 2017  [57] | Swedish MS registry data study including 14545 PwMS and 43,635 controls | Sweden | Investigation of the association between epilepsy and clinical features of MS | The cumulative incidence of epilepsy was 3.5% (95% CI 3.17-3.76) in patients with MS and 1.4% (95% CI 1.30-1.52) in controls (RR 2.5, 95% CI 2.19-2.76) |
| Martínez-Lapiscina et al, 2013  [58] | Cohort study of 428 PwMS  No control group | Spain | Evaluation of the prevalence, clinical and paraclinical features of epileptic  attacks in PwMS and MS-specific risk factors for  epileptic seizures | Thirteen patients (3%) were identiﬁed as having epileptic attacks. Ten patients (77%) experienced focal seizures, half of whom had conﬁrmed secondary generalization. MS patients with seizures had a significantly higher number of cortical and juxtacortical lesions on T2-  weighted/fluid attenuation inversion recovery magnetic resonance imaging than control group OR = 2.6 (95% CI, 1.0–6.5, p=0.047).  No control group was present for comparison |
| Benjaminsen et al, 2017  [59] | Retrospective chart review of 431 PwMS (19 with epilepsy and 411 without) | Norway | Assessment of records for evidence of comorbid seizures and MS | The frequency of active epilepsy among MS patients in Nordland was 3.2%, approximately 4.5 times higher than in the general Norwegian population. In patients with relapsing-remitting MS  (RRMS) at onset and active epilepsy (n = 10), 70% had converted to secondary progressive (SPMS) at prevalence date, compared to only 35% of those without active epilepsy (p = 0.02).  The MS-epilepsy group was compared with the MS without epilepsy as a control |
| Allen et al, 2013  [60] | Retrospective review of two datasets of linked statistical hospital admission records covering the Oxford Record Linkage Study area (ORLS, 1963-1998) and all England (1999-2011). | UK | Calculated the rate of occurrence of hospital admission for epilepsy in people after admission for MS, compared with equivalent rates in a control cohort, and expressed the results as a relative RR. | The RR for hospital admission for epilepsy following an admission for MS was significantly high at 4.1 (95% CI 3.1-5.3) in the ORLS and 3.3 (95% CI 3.1-3.4) in the all-England cohort. The RR for a first recorded admission for epilepsy 10 years and more after first recorded admission for MS was 4.7 (2.8-7.3) in ORLS and 3.9 (3.1-4.9) in the national cohort. The RR for the converse-MS following hospitalization for epilepsy-was 2.5 (95% CI 1.7-3.5) in the ORLS and 1.9 (95% CI 1.8-2.1) in the English dataset.MS and epilepsy occur together more commonly than by chance. |
| Viveiros et al, 2011  [61] | Retrospective chart review of 160 PwMS  No control group | Brazil | Characterization of the prevalence and clinical, radiological and electroencephalographic characteristics of epileptic seizures in PwMS | Of 160 cases analyzed, 5 (3.1%) had suffered epileptic seizures. Analysis of serial studies of brain MRI and EEG enabled a causal relationship to be established between the diagnosis of mesial hippocampal sclerosis and partial control of the convulsive crises in one of the patients, leading to a lower prevalence of 2.5%. No control group was present for comparison |
| Uribe-San-Martín, et al 2013  [62] | Retrospective chart review of 310 PwMS, 10 with epilepsy | Chile | Assessment of records for evidence of comorbid seizures and MS | Of 310 MS patients, ten had the diagnosis of epilepsy (3.2%) and were younger and had an earlier onset of symptoms of MS compared to the group without epilepsy (p=0.04). Patients with poor epilepsy control (frequent seizures or development of status epilepticus) had lower brain volumes (p=0.01) and worse cognitive performance (p=0.03). The MS-epilepsy group was compared with the MS without epilepsy as a control |
| Marrie et al, 2016  [13] | Population-based study of 23,382 PwMS and 116,638 matches from the general population | Canada | Prevalence of comorbidity in PwMS from administrative health data from 4 Canadian provinces, including British Columbia, Manitoba, Quebec, and Nova Scotia. | Crude prevalence of comorbidity in MS at diagnosis compared to matched controls. Hypertension (15.2% vs 12.9%, RR 1.17 (95% CI 1.13─1.21, p<,0.0001); diabetes (5.69% vs 4.86%, RR 1.17 (95% CI 1.10─1.24, p<0.0001); IHD (6.50% vs 5.01, RR 1.30 (95% CI 1.23─1.37, p<,0.0001); fibromyalgia (1.31% vs 0.46%, RR 2.87, 95% CI 2.49 ─3.30, p<0.0001); IBD (0.56% vs 0.30%, RR 1.68, 95% CI 1.38─2.04 p<0.0001). Chronic lung disease (12.1% vs 9.14%, RR 1.34, 95% CI 1.29─1.39); epilepsy (1.93 vs 0.89%, RR 2.18, 95% CI 1.95, 2.43, p<0.0001; depression (19.1% vs 9.38%, RR 2.04, 95% CI 1.97─2.10, p< 0.0001); Anxiety (11.1% vs 6.89%, RR 1.61, 95 CI% 1.54─1.68, p<0.0001; bipolar disorder (3.15% vs 1.69%, RR 1.86, 95% CI 1.71─2.02 p<0.0001; schizophrenia (1.07% vs 0.81%, RR 1.32, 95% CI 1.15─1.52, p<0.0001) in PwMS vs control population, respectively. |
| Etemadifar et al, 2012  [63] | Retrospective population-based study of 117 patients with early onset MS (EOMS) and 3405 non-early onset PwMS | Iran | Clinical/paraclinical details and frequency of epileptic seizures in Iranian early onset MS (EOMS) patients registered with the IMSS | The frequency of epilepsy in EOMS patients (8.5%, 10/117) was significantly greater (P < 0.001) than that of non-EOMS cohort (2.0%, 71/3405) |
| Etemadifar et al, 2013  [64] | Retrospective population-based study on the total Isfahan cohort of 3,522 Iranian PwMS patients, we looked for EP/MS patients and compared their features with 1,665 non-EP/MS cases. | Iran | Assessment of the duration of EP after MS diagnosis, and EP patterns in different types of EP | Among 81 (2.3 %) eligible cases, EP occurred within a mean duration of 5.6 ± 5.4 years after the development of MS in 64 cases (79 %); EP occurred at MS onset as the presenting symptom in five cases (6.2 %); and by a mean duration of 4.3 ± 4.3 years prior to onset of MS in 12 patients (14.8 %). The overall mean ages at MS onset and at the first seizure episode were 28.5 ± 11.2 years and 32.5 ± 14.0 years, respectively. Regarding the age at onset of MS, 12.3 % of EP/MS patients were classified as early-onset; 81.5 % as adult-onset; and 6.2 % as late-onset. Such frequencies were statistically different (P < 0.0001) from those of the 1,665 control non-EP/MS patients (5.9; 93.0 and 1.1 %, respectively). Regarding the pattern of MS, EP/MS patients were classified as relapsing-remitting, secondary progressive, and primary progressive in 60.5, 25.9, and 13.6 %, respectively. This configuration differed (P < 0.0001) from that of non-EP patients (87.9; 6.3 and 5.7 %, respectively). Results are suggestive of differences between EP/MS and non-EP/MS cases as regards the proportion of MS patterns and age-at-onset |

### Table 4: Restless leg syndrome

| Reference | Study type | Region | Measure | Outcome |
| --- | --- | --- | --- | --- |
| Lebrato Hernández et al, 2019  [65] | Cohort study in 120 PwMS.  No control subjects | Spain | Prevalence of RSL in MS cohort and relationship with relapse and pain and psychiatric conditions | The prevalence rate of RLS was 23.3%. MS progression time was significantly shorter in patients with RLS (P = 0.001). A recent relapse, and symptoms of anxiety, depression, and neuropathic pain were significantly associated with risk of RLS (P = 0.001, P < 0.001, P < 0.001, and P = 0.001, respectively).  No control group was present for comparison |
| Bruno et al, 2014  [66] | Case-controlled cohort study in 152 PwMS and 431 controls | Sicily | RLS prevalence and association with spinal cord lesions | A significantly higher prevalence of RLS amongst MS patients (14.5%) compared with controls (6.0%) was detected, corresponding to an almost threefold increased risk (OR 2.7, 95% CI 1.4-5.0) of developing RLS. Spinal cord lesions in MS patients were associated with a higher risk of RLS (odds ratio 3.7, 95% CI 1.1-13.5). |
| Minár et al, 2016  [67] | Cross sectional study in 200 PwMS.  No control subjects | Slovakia | Determination of RLS prevalence and risk factors | 26% were RLS-positives (95% CI 20−32%). From positive patients, 44% had negative family history for RLS, and developed secondary RLS after onset of MS. Compared to RLS-negatives, the positives had significantly higher prevalence of spinal cord lesions (p=0.01). Presence of spinal pathology related to higher risk of RLS development (OR=3.846, 95% CI 1.30−11.35).  No control group was present for comparison |
| Schürks and Bussfeld, 2013  [68] | Systematic review and meta-analysis of MS cases and controls.  24 studies | USA/  Canada, Spain/ Italy/ Portugal, Brazil, Macedonia,  France/ Poland/ Germany/Czech Republic, Turkey,  Iran | RLS prevalence and risk factors | RLS prevalence amongst patients with MS ranged from 12.12% to 57.50% and from 2.56% to 18.33% amongst people without MS. Heterogeneity amongst studies was high (RLS prevalence in patients with MS I(2) =94.4%; RLS prevalence amongst people without MS I(2) =82.2%). MS is associated with four-fold increased odds for RLS (pooled OR=4.19, 95% CI 3.11−5.66). |
| Sorgun et al, 2015 [69] | 91 PwMS and 40 patients in a control group (headache, essential tremor, and benign positional paroxysmal vertigo) were studied | Turkey | Investigation of RLS prevalence, neurological examination and Kurtzke functional system scores were calculated | Sixteen (17.6%) of the patients with MS and 1 (2.5%) patient in the control group had RLS. The prevalence of RLS was higher in patients with MS, compared to the control group (P = 0.018). Among the patients with MS, none of them suffered from RLS before the onset of MS, whereas sixteen patients (17.6%) suffered RLS after the onset of MS. There was no significant relationship between functional system involvement and the presence of RLS. The prevalence of RLS was higher in MS patients than it was in the control group (P=0.018). No association was found between RLS and functional system involvement in MS patients. |
| Carnero Contentti et al, 2019  [70] | Cross- sectional study in 189 PwMS and 238 control subjects | Argentina | Investigation of RLS prevalence and risk factors | Clinically significant RLS (csRLS; ie, symptoms present ≥2 days per week) frequency was 19.4% in patients with MS versus 4.2% in controls (OR = 5.37, P<0.00001). Longer MS duration and presence of anxiety, depression, insomnia, and smoking cigarettes were all significantly associated with RLS. |
| Ning et al, 2018  [71] | Systematic review and meta-analysis of 7286 PwMS and 2583 control subjects | Inside vs outside of Asia | Investigation of the association between MS and RLS | Pooled RLS prevalence among MS patients of various ethnicities was 26%, and prevalence was lower in Asia (20%) than outside Asia (27%). Prevalence was higher among cross-sectional studies (30%) than among case-control studies (23%). RLS prevalence was higher among female than male MS patients (26% vs. 17%), and it was higher among MS patients than among healthy controls (OR 3.96, 95% CI 3.29-4.77, p < 0.001). RLS prevalence ranged from 13.3% to 57.5% in MS patients, while it ranged from 2.5% to 18.3% in controls. |
| Liu et al, 2015  [72] | Case-controlled cross-sectional study in 695 PwMS and 603 control subjects | China | Investigation of RLS incidence in participants with or without MS. We further assessed sleep quality in all the participants. | Higher prevalence of RLS among patients with MS (24.6%) compared to healthy controls (8.0%) (odds ratio [OR], 3.8; P < 0.001). MS and RLS were more likely to suffer from sleep complaints compared to patients with MS without RLS |
| Miri et al, 2012  [73] | Cross-sectional study in 205 MS patients.  No control group | Iran | Assessment of the frequency of RLS and its associated factors in patients with MS. | 57 patients (27.8%) met the RLS diagnostic criteria. In 90.1% of the patients, the onset of RLS was simultaneous with or followed by MS onset. In patients suffering from RLS, there was no significant difference between patients with and without RLS in respect of age, gender, disease  duration, and MS pattern but there is greater complaint of insomnia (p=0.03).  No control group was present for comparison |
| Shaygannejad et al, 2013  [74] | Cross-sectional study in 126 PwMS and 126 matched control subjects | Iran | Investigation of RLS prevalence and risk factors | The frequency of RLS in the MS patients was significantly higher in MS group (82, 65.1%) than in the control group, (16, 12.7%) (p< 0.001). MS patients and higher EDSS score had more RLS symptoms (p<0.005) |

### Table 5: Migraine, fibromyalgia, ocular and olfactory comorbidities

| Reference | Study type | Region | Measure | Outcome |
| --- | --- | --- | --- | --- |
| Pakpoor et al, 2012  [75] | Systematic review and meta-analysis of 1864 PwMS and 261,563 control subjects. | NA | Investigation of migraine prevalence in PwMS | Migraine was more common in MS patients than controls: the overall OR upon inclusion of all 8 studies was 2.60 (95 CI 1.12–6.04), significant statistical heterogeneity was identified (I2 =97% Chi2 =247.10, p<,0.00001). Subgroup analyses of studies investigating migraine with and without aura showed a significant co-morbid association between MS and migraine without aura (OR=2.29, 95% CI 1.14–4.58)) without any significant heterogeneity |
| Bazelier et al, 2011  [76] | Population-based cohort study of 5576 PwMS cases, each matched to six patients without MS (controls) | UK | To evaluate whether multiple sclerosis (MS) is associated with risk of cataract or glaucoma. | MS patients had no overall increased risk of cataract, adjusted (adj.) HR 1.15 (95% CI 0.94−1.41) or glaucoma, adj. HR 1.02 (95% CI 0.78−1.33). Risk of cataract (adj. HR 2.45 (95% CI 1.56−3.86)) and glaucoma (adj. HR 1.70 (95% CI 1.01−2.86)) was significantly greater in patients < 50 years, particularly in men < 50 years: cataract, adj. HR 4.23 (95% CI 2.22−8.05) and glaucoma, adj. HR 2.76 (95% CI 1.28−5.93).This is the first study which showed that the risk of cataract and glaucoma is elevated in MS patients younger than 50 years, particularly men. |
| Villani et al, 2012  [77] | Cohort study in 205 PwMS and 63 control subjects without with migraine | Italy | Prevalence of migraine in MS, determinant and treatment of migraine and MS. BDI, STAI-1 and 2, TAS-20, MIDAS, EDSS and FSS | 102 (49.8 %) were diagnosed as affected by comorbid migraine. About one-third of MS patients with comorbid migraine have asked the attending neurologist a specific anti-migraine treatment. Despite this, only few MS patients (10.8 %) reported a prior use of prophylactic drugs, and even fewer (2.9 %) took triptans as pain killers; these proportions were significantly lower when compared with those of a control group of 63 migraineurs subjects without MS (p < 0.0001 for both comparison). |
| Silva et al, 2012  [78] | Cohort study in 153 PwMS and 165 healthy control subjects | Portugal | Assessment of odor identification capacities in multiple sclerosis (MS). | Patients with MS (11.1%) were more impaired on the B-SIT than HC participants (3%, p=0.007). The frequency of impairment was higher for patients with secondary progressive (SPMS; 11/16, 68.8%) than relapsing–remitting (RRMS; 4/121, 3.3%) or primary progressive (2/16, 12.5%) courses (p<0.001). |
| Krökki et al, 2014  [56] | Cohort study of 491 PwMS diagnosed 1990 and 2010  No control group | Finland | Measurement of the prevalence of one or more neurological condition | One or more neurological comorbid disease was present in 17.1% of patients (n=84). The prevalence of epilepsy in MS patients was 4.7%, which is greater than that in the general population. Migraine was significantly more common in women with a benign MS course when compared to other types of MS (p=0.046). A significant association between peripheral nervous system disorders and primary progressive MS was found (p=0.027).  No control group was present for comparison |
| Sahai-Srivastava et al, 2016  [79] | Cross-sectional study of 233 PwMS  No control group | USA (multi-ethnic population) | Prevalence and type of headache across a multiethnic MS population, and relationship between MS related clinical factors and migraine | Chronic migraine was more common among Hispanics (82%) than Whites (18.2%) (p=0.012). Headache impact on daily life, measured by HIT-6 score (p=0.006) and PHQ-9 score (p=0.004) were significantly higher in the public sector. After controlling for income and education, female gender (OR 2.59, 95% CI 1.31−5.13) and ambulatory disability were found to be more likely to suffer from migraines. Headache, especially migraine is common among MS patients regardless of socio-economic status and treatment setting.  No control group was present for comparison |
| Lincoff et al, 2017  [80] | Retrospective chart review of 30 PwMS and 30 matched control subjects | USA | Determination of whether MS is associated with lower intraocular pressure (IOP) compared with individuals without MS. | There was a significant effect of presence of MS on IOP accounting for 53% variability in mean IOP (F(1,55) = 60.7; p < 0.001) when compared with the control group. This study demonstrated that IOP was significantly lower in patients with MS compared with controls. |
| Newland et al, 2015  [30] | Cross-sectional study of 339 PwMS compared with normal population | USA | Comparison of the % of health conditions between  PwMS and U.S. national norms | Rates of depression (55% vs 3%, p<0.0001), cancer (15% vs 9%, P=0.01), migraine (37% vs 17%, b) and arthritis (32% vs 25%, p=0.003) are higher in MS than the US norm |
| Kister et al, 2010  [81] | Cross-sectional study  in 94 PwMS with 73 headache-free MS patients and the American Migraine Prevalence and Prevention (AMPP) study population | USA | Assessed the demographic proﬁles, headache features and clinical characteristics of MS patients attending a MS clinic using a survey based on the American Migraine Prevalence and Prevention (AMPP) study | The relative frequency of migraine was threefold higher than in MS population controls both for women [55.7 vs. 17.1%; prevalence ratio (PR) = 3.26, p<0.001] and men (18.4 vs. 5.6%; PR = 3.29, p<0.001). |
| Kowalec et al, 2017  [12] | 2-year prospective multicenter cohort study in 885 PwMS  No control group | Canada | Cross-sectional assessment of comorbidities and relapses. Comorbidities were recorded using questionnaires, and relapses were captured from medical records at each visit. | Anxiety (40.2%), depression (21.1%), hypertension (17.7%), migraine (18.1%), and hyperlipidemia (11.9%) were the most prevalent comorbidities.  Migraine and hyperlipidemia were associated with increased relapse rate (adjusted rate  ratio 1.38; 95% CI 1.01–1.89 and 1.67, 95% CI 1.07–2.61, respectively)  No control group was present for comparison |
| Marrie et al, 2012  [82] | Population based study with 4192 PwMS and 20, 940 persons from the general population | Canada | Case definition and prevalence of fibromyalgia in PwMS using administrative health data | In 2005, the age-standardized prevalence of fibromyalgia was 6.82% (95% CI 5.91─7.72) in the MS population and 3.04% (95% CI 2.7−3.32) in the GP. After adjustment for age, sex and year, the incidence of fibromyalgia was 44% higher in the MS than the GP (IRR 1.44, 95% CI 1.01−2.07). The incidence of fibromyalgia increased slightly over time in both populations. The incidence and prevalence of fibromyalgia are higher in the MS population than the general population. |
| Jordy et al, 2016  [83] | Cross sectional study in 100 PwMS patients and 100 healthy control patients | Brazil | Assessment of olfactory function using the Connecticut test and verify correlations between olfactory alteration, disease duration and EDSS. | Thirty-two percent of patients showed alterations, compared with 3% in the healthy control group. Patients having EDSS above 4, showed a 5.2-times increased risk of dysfunction. Patients over 38 years of age have a 2.2-times increased risk over younger patients (p=0.001, 95% CI 1.8–15.1). |

### Table 6: Autoimmune conditions

| Reference | Study type | Region | Measure | Outcome |
| --- | --- | --- | --- | --- |
| Liu et al, 2019  [84] | Systematic review and meta-analysis. The case-control and cross-sectional studies: 18,456 PwMS and 870,149 controls; the two cohort studies: 25,187 PwMS and 227,225 control subjects | NA | Prevalence of psoriasis in MS (odd and risk ratios) | MS was associated with increased odds (OR 1.29; 95% CI 1.14-1.45) and risk for psoriasis (HR 1.92; 95% CI 1.32-2.80). Patients with MS display both increased prevalence and incidence of psoriasis. |
| Lorefice et al, 2018  [85] | Cohort study with 286 PwMS (comparing 78 with and 208 without comorbid autoimmune disease)  No control group. | Italy | Presence of autoimmune disease and relationship to T1-weighted MRI structural images. | 30 (10.5%) subjects had type 1 diabetes (T1D), and 53 (18.5%) had autoimmune thyroiditis (AT) and 4 (0.1%) with celiac disease. Association between T1D and lower gray matter (GM) (p = 0.038) and cortical GM (p = 0.036) volumes, independent from MS clinical features and related to T1D duration (p < 0.01).  No control group was present for comparison |
| Annunziata et al, 2010  [86] | Cross-sectional observational study in 440 PwMS of whom 230 took DMT and 210 were DMT naïve | Italy | Structured neurologist-administered questionnaire investigating the presence of clinical features of Sjogren’s syndrome (SS) at the time of interview and at the time of MS onset | 28 of 230 (12%) patients receiving treatment (DMTs+) and 14 of 210 (6.6%) treatment-naive patients (DMTs) showed clinical features of SS (p = 0.053). SS syndrome were significantly associated with higher EDSS scores (P = 0.018), a low frequency of gadolinium-enhanced MRI-positive lesions (P = 0.018) and cerebral disturbances (p = 0.001). |
| Fanouriakis et al, 2014 [87] | Cohort study of 819 PwMS and 728 with systemic lupus erythematosus (SLE) | Greece (Crete) | Investigation of the coexistence of SLE and MS using clinical, laboratory, and neuroimaging findings | 9 patients who fulfilled the diagnostic criteria for both SLE and MS, corresponding to a prevalence rate of 1.0-1.2% in each cohort. All patients were women, with an average age at SLE diagnosis of 42.1 years (range: 34-56 years). The diagnosis of SLE preceded the development of MS in five patients, with a time lag ≤ 5 years in four of them. Occurrence of both diseases in the same individual is rare, corroborating data that suggest distinct molecular signatures. SLE and MS coexistence was not associated with a severe phenotype for either entity. |
| Deretzi et al, 2010  [88] | Prospective case-control study in 3112 PwMS and 1580 control subjects | Greece | Relationship between immunomodulatory treatment and the appearance of additional autoimmune disorders in PwMS | There was an increase in longitudinally affected MS families, MS family members and coexistent additional autoimmune disorders compared with respective findings at the baseline observation. Comparison analysis between two time point observations (after a mean 7.1 +/- 2.2 years) for each autoimmune disorder in overall MS family members revealed increased rates for longitudinal autoimmune Hashimoto's thyroiditis, Graves' disease, insulin-dependent diabetes mellitus, psoriasis and vitiligo (p = 0.02, p = 0.006, p = 0.0004, p = 0.05, and p = 0.05, respectively). Some 145 newly developed, longitudinally definite autoimmune cases were recognized in multiplex plus simplex MS families; 116 (80%) of these disorders were observed in patients with MS treated with immunomodulatory medications, and 68 of these 116 (58.6%) cases exhibited baseline positive autoreactive antibodies. Binary logistic regression analysis revealed that immunotherapy predisposes to autoimmunity (OR 2.8, p < 0.001) independently of the presence of baseline autoantibodies and patients' gender. There is a longitudinally increased frequency of additional autoimmune disorders among MS family members, probably related to immunomodulatory therapy. |
| Egeberg et al, 2015  [89] | All Danish citizens aged ≥ 18 years from 1 January 1997 to 31 December 2011 were identified by linkage of nationwide registries at the individual level.  58,628 and 9,952 cases of mild and severe psoriasis, respectively, and 9,713 cases of MS | Denmark | Estimation of incidence rate ratios (IRRs) adjusted for age, gender, socioeconomic status, smoking, medication, comorbidity, and UV phototherapy by Poisson regression. | Incidence rates of MS per 10,000 person-years for the reference population, mild psoriasis, and severe psoriasis were 1.78, 3.22, and 4.55, respectively. Adjusted IRRs of MS were 1.84 (95% CI 1.46−2.30) and 2.61 (95% CI 1.44−4.74) in mild and severe psoriasis, respectively. The |
| Chouhfeh et al, 2015  [90] | Registry study following 1792 PwMS (with 5 years follow-up). PwMS were stratified by presence of AID and DMT use | USA | Prevalence of AID and association with DMT use | 1478 patients (82.1%) did not have an AID. The most frequently reported AID in DMT- group were thyroid disease (n=11, 33.3%), IBS (n=5, 15.2%). The most reported AID in the DMT+ group were thyroid disease (n=95, 33.8%) and IBS (n=68, 24.2%). Duration between first MS symptom onset and the initial reported occurrence of a comorbid AID was significantly shorter in the DMT user group (192 months) compared to the DMT naïve group (262 month, p=0.002). |
| Zivadinov et al, 2016  [91] | Retrospective chart review in 815 PwMS, 241 with comorbidities and 574 without comorbidities | USA | Determination of the association of comorbidities with MR imaging disease severity outcomes in MS | Two hundred forty-one (29.6%) study subjects presented with comorbidities. Thyroid disease had the highest frequency (n = 97, 11.9%), followed by asthma (n = 41, 5%), type 2 diabetes mellitus (n = 40, 4.9%), psoriasis (n = 33, 4%), and rheumatoid arthritis (n = 22, 2.7%). The presence of comorbidities in patients with MS is associated with brain injury (reduced brain volume) on MR imaging (p<0.05). Psoriasis, thyroid disease, and type 2 diabetes mellitus comorbidities were associated with more severe nonconventional MR imaging outcomes. |
| Guido et al, 2017  [92] | Large single center, retrospective cross-sectional study in 566,122 individuals recorded in a medical database | USA | Determination of an association for MS and  psoriasis in a large, urban, single center population. Data for patients who had psoriasis and/or MS (using ICD-9 codes 696.1 and 340, respectively | 5,097 persons were diagnosed with psoriasis, of whom 417 were also diagnosed with psoriatic arthropathy, and 1,829 were diagnosed with MS. Of these, 26 were diagnosed with both psoriasis and MS of whom 5 were also diagnosed with psoriatic arthropathy. A significant association between psoriasis and MS was detected after adjusting for confounding variables of sex, age, psoriatic arthritis and TNF-α agent exposure (OR=1.52, 95% CI 1.01 −2.29; p=0.04). |
| Newland et al, 2015  [30] | Cross-sectional study of 339 PwMS compared with normal population | USA | Comparison of the % of health conditions between  PwMS and U.S. national norms | Rates of depression (55% vs 3%, p<0.0001), cancer (15% vs 9%, P=0.01), migraine (37% vs 17%, b) and arthritis (32% vs 25%, p=0.003) are higher in MS than the US norm |
|  |  |  |  |  |
| Marrie et al, 2017  [93] | Case controlled cohort study of 4911 PwMS to 23,274 control subjects | Canada | Evaluation of data for patients who had psoriasis and/or MS (using ICD-9/10 codes were extracted for | In 2008, the crude incidence of psoriasis per 100,000 person-years was 466.7 (95% CI 266.8−758.0) in the MS population, and 221.3 in the matched population (95% CI 158.1−301.4). The crude prevalence of psoriasis per 100,000 persons was 4666.1 (95% CI 3985.2−5429.9) in the MS population, and 3313.5 (95% CI 3057.4−3585.3) in the matched population. The incidence and prevalence of psoriasis rose slightly over time. After adjusting for sex, age at the index date, socioeconomic status and physician visits, the risk of incident psoriasis was 54% higher in the MS population (HR 1.54, 95% CI: 1.07−2.24).Psoriasis incidence and prevalence are higher in the MS population than in the matched population. |
| Marrie et al, 2016  [13] | Population-based study of 23,382 PwMS and 116,638 matches from the general population | Canada | Prevalence of comorbidity in PwMS from administrative health data from 4 Canadian provinces, including British Columbia, Manitoba, Quebec, and Nova Scotia. | Crude prevalence of comorbidity in MS at diagnosis compared to matched controls. Hypertension (15.2% vs 12.9%, RR 1.17 (95% CI 1.13─1.21, p<,0.0001); diabetes (5.69% vs 4.86%, RR 1.17 (95% CI 1.10─1.24, p<0.0001); IHD (6.50% vs 5.01, RR 1.30 (95% CI 1.23─1.37, p<,0.0001); fibromyalgia (1.31% vs 0.46%, RR 2.87, 95% CI 2.49 ─3.30, p<0.0001); IBD (0.56% vs 0.30%, RR 1.68, 95% CI 1.38─2.04 p<0.0001). Chronic lung disease (12.1% vs 9.14%, RR 1.34, 95% CI 1.29─1.39); epilepsy (1.93 vs 0.89%, RR 2.18, 95% CI 1.95, 2.43, p<0.0001; depression (19.1% vs 9.38%, RR 2.04, 95% CI 1.97─2.10, p< 0.0001); Anxiety (11.1% vs 6.89%, RR 1.61, 95 CI% 1.54─1.68, p<0.0001; bipolar disorder (3.15% vs 1.69%, RR 1.86, 95% CI 1.71─2.02 p<0.0001; schizophrenia (1.07% vs 0.81%, RR 1.32, 95% CI 1.15─1.52, p<0.0001) in PwMS vs control population, respectively. |
| Marrie et al, 2012  [94] | Case-matched population study in 4,192 PwMS and 20,940 matches from the general population | Canada | Classification of Disease-9/10 codes and prescription claims, compared them to medical records and applied them to estimate the incidence and prevalence of autoimmune thyroid disease (AIT). | In 2005, the age-adjusted prevalence of AIT was 9.51% (95% CI 8.46−10.6) in the MS population and 8.56% (95% CI 8.11−9.02) in the general population. The age-adjusted incidence of AIT per 100,000 persons per year was 422.8 (95% CI 204.4−641.3) in the MS population and 407.7 (95% CI 308.5−506.9) in the general population. From 1996 to 2005, the prevalence of AIT rose in both populations. Administrative data can be used for surveillance of AIT in MS. The incidence and prevalence of thyroid disease are similar in the MS and general populations. |
| Farez et al, 2014  [95] | Case-controlled study with 211 PwMS and 211 control subjects | Argentina | Prevalence of AID in MS compared with controls | 28% of MS patients reported at least one autoimmune comorbidity, with no significant differences with respect to healthy controls (32%, 𝜒2 0.45). Most frequently AIS in MR was thyroid disease (9%), asthma (6%), atopic dermatitis (5%), and type I diabetes (3%). No differences between MS patients and controls were reported. Finally, presence of one or more autoimmune disorders did not increase risk of developing MS (OR 0.85, 95% CI 0.6–1.3) nor did age of diagnosis or gender show any effect on autoimmune disease rates. |
| Tettey et al, 2016  [14] | Prospective cohort of 198 PwMS | Australia | Evaluation of specific doctor-diagnosed comorbidities in PwMS versus the 207 general population prevalence | The age-standardized prevalence of hypertension (21.21% vs 9.43%), dyslipidemia (14.65% vs 5.72%), asthma (18.69% vs9.93%), psoriasis (7.58% vs 2.29%), eczema (13.13% vs 0.92%) and anemia (12.63% vs 1.83%) were significantly higher in the MS cohort compared to that in the general Australian population (p≤0.001.  For relapse analyses, rheumatoid arthritis and anemia were associated with more than threefold (HR 3.70, 95% CI 1.80─7.58, p=0.001) and twofold (HR 2.04, 95% CI 1.11─3.74, p = 0.022) increased risk of subsequent relapse respectively. The prevalence of some comorbidities was higher in MS patients and associated with greater disability and relapse risk. |
| Tseng et al, 2016  [96] | Nationwide matched cohort study in 1456 PwMS and 10362 control subjects | Taiwan | Prevalence and possible relationship of RA in MS in patients versus controls | Patients with MS had a higher incidence of rheumatoid arthritis (age-adjusted standardized incidence ratio: 1.72, 95% CI 1.01−2.91). There was a positive correlation in being diagnosed with rheumatoid arthritis in patients previously diagnosed with MS when stratified by sex and age. The strength of this association remained statistically significant after adjusting for sex, age, and smoking history (hazard ratio: 1.78, 95% CI 1.24−2.56, P = 0.002) |
| Miron et al, 2017  [97] | Retrospective case-control study of 3456 PwMS (those with psoriasis identified and compared with cohort of MS only patients) | Israel | Clinical and demographical characteristics and MS progression-related outcomes in patients whose follow-up exceeded 5 years were analyzed and compared to those of a matched control cohort of MS-only (MSO) patients | Forty-five (1.3%) MS patients had psoriasis comorbidity. Psoriasis preceded MS in 35 (78%) cases. Patients with psoriasis onset preceding relapsing-remitting MS (RRMS) had slower progression of disease compared to MSO patients, as manifested by a longer time to second relapse (P <0.01) and a longer time to significant neurological disability scores (P< 0.03). |
| Fellner et al, 2014  [98] | Case control study of 214 PwMS and 192 (headache) control subjects | Israel | Medical chart review for existence and type of psoriasis | 9/214 (4.21%) in MS group versus 1/192 (0.52%) in the control group had psoriasis. (p=0.021) OR=8.39, 95% CI 1.05−66.81 |
| Tseng et al, 2016  [96] | Nationwide matched cohort study in 1456 PwMS and 10362 control subjects | Taiwan | Prevalence and possible relationship of RA in MS in patients versus controls | Patients with MS had a higher incidence of rheumatoid arthritis (age-adjusted standardized incidence ratio: 1.72, 95% CI 1.01−2.91). There was a positive correlation in being diagnosed with rheumatoid arthritis in patients previously diagnosed with MS when stratified by sex and age. The strength of this association remained statistically significant after adjusting for sex, age, and smoking history (hazard ratio: 1.78, 95% CI 1.24−2.56, P = 0.002) |
| Kang et al, 2010  [16] | Cohort study in 898 PwMS and 4490 control subjects | Taiwan | Evaluation of 30 comorbid medical conditions from insurance claims | PwMS were more likely to have systemic lupus erythematosus (OR = 26.9, 95% CI 10.3−70.3), depression (OR = 6.9, 95% CI 5.3─8.9), peripheral vascular disorders (OR = 6.6, 95% CI 4.0−11.0), deficiency anemias (OR = 4.9, 95% CI 2.8−8.7), rheumatoid arthritis (OR = 4.8, 95% CI 2.9−8.1) and fluid and electrolyte disorders (OR = 4.8, 95% CI 2.8−8.3) than the matched controls. Patients with MS had higher risk of multiple medical comorbidities compared to a matched control group in an ethnic Chinese population. |

### Table 7: Cancer

| Reference | Study type | Region | Measure | Outcome |
| --- | --- | --- | --- | --- |
| Hongell et al, 2019  [99] | Nested case control study in 1074 PwMS and 10740 control subjects | Finland | Assessment of cancer risk for each cancer diagnosis in PwMS | A total of 61 (5.7%) of the MS patients and 757 (7.0%) of the controls were diagnosed with cancer during the study period. The overall risk of cancer in the MS cohort did not signiﬁcantly diﬀer form the controls (OR 0.80, 95% CI 0.6–1.0, p=0.092). |
| Nørgaard et al, 2019  [100] | MS registration study in 10,752 PwMS vs expected in general population | Denmark | Cancer incidence and cancer-speciﬁc mortality in MS patient | Among 10,752 MS patients, 5.76 incident cancers per 1,000 person-years were identiﬁed. The standardized incidence ratio was 0.98 (95% conﬁdence interval [CI], 0.90–1.06) for any cancer, 0.99 (95% CI 0.84–1.15) for non-melanoma skin cancer, and 0.98 (95% CI, 0.81–1.18) for female breast cancer. For malignant melanoma, the standardized incidence ratio was 1.51 (95% CI 1.13–1.98) for the entire period (1995–2015) but 1.16 (95 CI 0.62–1.99) for 2005–2015. |
| Handel & Ramagopalan, 2011  [101] | Meta-analysis of five population-based cohort studies of 45,032 PwMS  No control group | Europe (UK, Sweden, France, Norway, Denmark) | Investigation of cancer risk in PwMS | A small but signiﬁcant decrease in cancer risk for patients with MS was detected (OR 0.92, 95% CI 0.87−0.97, p=0.004).  No control group was present for comparison |
| Moisset et al, 2017  [102] | Case-control study, using a postal survey of 1107 PwMS and 1568 control subjects (neurology outpatients) | France | Evaluation of lifetime cancer prevalence in a large cohort of MS patients relative to appropriate controls | Among the MS patients, 7.32% had ever presented with a cancer, whereas 12,63% of the controls had, leading to a bootstrap matched odds ratio (OR) of 0.63; 95% CI 0.57–0.70. Although only exploratory, the use of DMT (immunomodulators or immunosuppressants) did not appear to increase this risk (p = 0.42). The disease course also did not affect cancer prevalence |
| Lebrun et al, 2011  [103] | Population-based study in 22,563 PwMS | France | Evaluation of cancer incidence in PwMS who had and had not received immunomodulator (IM) and immunosuppressant (IS) | A total of 9,269 patients (44.1%) had a history of DMT (52% IM; 18% IS; 30% both). There was no increased risk of cancer among patients treated exclusively with IM. IS treatment (P = 0.043) and the duration of exposure (P<0.001) signiﬁcantly increased the risk of cancer, especially skin cancer, as observed in other autoimmune diseases |
| D’Amico et al, 2019  [104] | Population-based cohort study in 2,730 PwMS, comparing patients who had received at least two DMTs to those who had none or no DMT  No control group | Italy | Calculation of age and sex speciﬁc SIR and RR of developing cancer in MS patients treated with at least two different DMTs compared to who received one or no treatment | 36 cancers were found. Global SIR was 1.18 (95% CI 0.78–1.58), with a signiﬁcantly higher risk in men with a range age of 20 to 50 years of 2.84 (95% CI 1.59–4.09) and in women over 50 years SIR= 1.82 (95% CI 1.08–2.55). RR of developing cancer was 1.99 (95% CI 1.14–3.45) in MS patients switching one DMT and 3.38 (95% CI 1.83–6.22) in who switched at least twice  No control group was present for comparison. |
| Ragonese et al 2017  [105] | Population-based cohort study in 531 PwMS vs expected in general population | Sicily | Cancer incidence was compared in MS and DMT treatment with the general population | Higher cancer risk in MS patients associated with previous DMT treatment (HR: 11.05; 95% CI 1.67−73.3; p=0.013 |
| Hajiebrahimi et al, 2016  [106] | Registry study in 19,330 PwMS with 193,458 matches from the general population | Sweden | Investigation of the risk of breast cancer risk in premenopausal MS patients | Overall risk of postmenopausal breast cancer was 13% higher among MS patients compared with women without MS (HR = 1.13, 95% CI 1.02–1.26). Stratified analyses showed that the risk was statistically significantly increased in women diagnosed between 1968 and 1980 and those who were diagnosed at age 65 or older age. There was a non-statistically significant risk only for stage 0–1 postmenopausal breast cancer (HR = 1.17, 95% CI 0.93–1.48). MS was not associated with premenopausal breast cancer. |
| Jick et al, 2015  [6] | Cohort study in 1713 PwMS.  No control group | UK | Assessment of lifetime incidence of comorbidity in PwMS | Following MS diagnosis, frequent comorbidities were infections (80 %), and depression (46 %). Incidence of chronic comorbidities recorded at any time in the database were: Chronic obstructive pulmonary disease (COPD) and asthma (20.0%) depression (45.8%) diabetes (5.4%) hypertension (14.8%) heart disease (3.9%) cancer (6.4%)  No control group was present for comparison. |
| Kingwell et al, 2013  [107] | Population-based observational study in 5146 PwMS were matched with up to 20 randomly selected MS controls | Canada | Associations between MS treatment exposure and overall or specific (breast, colorectal, lung and prostate) cancers were estimated | Exposure to IFNβ was not significantly different for cases and controls (OR 1.28; 95% CI 0.87−1.88). A non-significant trend towards an increased risk of IFNβ exposure in the breast cancer cases (OR 1.77; 95% CI 0.92−3.42), but no evidence of a dose-response effect; evidence of an increased cancer risk with exposure to IFNβ over a 12-year observation period |
| Kingwell et al, 2012  [108] | Cohort study in 6820 PwMS  No control group | Canada | Cancer risk and tumor size at MS diagnosis in a cohort of PwMS compared to the general population, also explored the inﬂuence of disease course | The standardized incidence ratio for all cancers was 0.86 (95% conﬁdence interval: 0.78–0.94). Colorectal cancer risk was also signiﬁcantly reduced (standardized incidence ratio: 0.56; 95% conﬁdence interval: 0.37–0.81). Tumor size was larger than expected in the cohort (p=0.04). Overall cancer risk was lower in patients with multiple sclerosis than in the age-, sex- and calendar year matched general population. The larger tumour sizes at cancer diagnosis suggested diagnostic neglect; this could have major implications for the health, well-being and longevity of people with multiple sclerosis.  No control group was present for comparison |
| Gaindh et al, 2016  [109] | Retrospective chart review of 9240 PwMS vs expected in general population | USA | Overall cancer risk. Self-reported cancers in MS patients were tested for associations with DMT use, family history of cancer and other factors | There were no significant differences in MS type, EDSS score, race or education between MS patients reporting cancer vs not. The prevalence of cancer was lower than expected in MS patients (p<0.001) |
| Etemadifar et al, 2017  [110] | Cohort study in 1718 PwMS.  No control group | Iran | Evaluation of prevalence and incidence ratio of expected cancer in MS population | MS significantly affects certain cancers in a protective or associative manner (all cancers SIR=0.95, 95% CI 0.80−1.10). All cancer rates, except breast cancer, cancers located in the nervous system, and lymphomas were reduced in cohort, suggesting that unregulated immune function may provide protective effects to MS patients against cancer  Further analysis resulted in an increased risk of breast cancer (SIR=1.77, 95% CI, 1.12−2.76), lymphoma (SIR=1.87, 95% CI, 1.64−2.20)] and cancers which are located in nervous system (SIR=2.30, 95% CI, 1.01−5.05)] and decreased risk of other cancers (SIR=0.40, 95% CI, 0.60−0.90] including: endocrine glands, bone, connective tissue, secondary and unspecified sites. |
| Sun et al, 2014  [111] | Population-based cohort study in 1292 PwMS and 5168 control subjects | Taiwan | Estimation of the inﬂuence of MS on cancer risk | Overall incidence rate of all cancer types was 1.71-fold higher in the MS cohort than in the comparison cohort (5.29 vs. 3.09 per 1000 person-years) with an adjusted HR of 1.85 (95% CI 1.26–2.74); but no signiﬁcant association between MS and overall cancer risk for those with any comorbidity. |

### Table 8: Metabolic disorders, dyslipidemia, diabetes

| Reference | Study type | Region | Measure | Outcome |
| --- | --- | --- | --- | --- |
| Wens et al, 2014  [112] | Cross-sectional study in 80 PwMS and 45 control subjects  [112] | Belgium | Determination of whether PwMS are at higher risk of impaired glucose tolerance (IGT) | The prevalence of impaired fasting glucose concentrations (17% vs 2%) and IGT (11% vs 0%) was higher in MS patients than control subjects (p<0.05) |
| Sicras-Mainar et al, 2017  [23] | Retrospective cohort study using electronic medical records with 222 PwMS.  No control group | Spain | MetS was defined using the National Cholesterol Education Program Adult Treatment Panel III. Patients were distributed into two groups according to the Expanded Disability Status Scale (EDSS) score: 0-3.5 and 4-10 | Depression (32.4%), dyslipidemia (31.1%), hypertension (23.0%) and obesity (22.5%) were the most common comorbidities. Overall MetS prevalence was 31.1% (95% CI: 25.0-37.2%). Patients with an EDSS ≥ 4.0 showed a significantly higher number of comorbidities (OR=2.2; 95% CI: 1.7-3.0; p<0.001).MS patients had a high prevalence of MetS.  No control group was present for comparison |
| Jick et al, 2015  [6] | Cohort study in 1713 PwMS.  No control group | UK | Assessment of lifetime incidence of comorbidity in PwMS | Following MS diagnosis, frequent comorbidities were infections (80 %), and depression (46 %). Incidence of chronic comorbidities recorded at any time in the database were: Chronic obstructive pulmonary disease (COPD) and asthma (20.0%) depression (45.8%) diabetes (5.4%) hypertension (14.8%) heart disease (3.9%) cancer (6.4%)  No control group was present for comparison. |
| Oliveira et al, 2014  [113] | Case controlled cross-sectional study in 110 PwMS and 175 control subjects | Brazil | Incidence of insulin resistance (IR) in MS patients and metabolic and inflammatory markers, oxidative stress, and disability were evaluated by EDSS | IR prevalence was verified in 40% of the patients with MS and in 21.1% of the control group (OR, 2.48; 95% CI, 1.47−4.21; p = 0.0006). Patients with the disease and IR showed higher EDSS (p = 0.03), interleukin (IL)-6 (p = 0.028), IL-17 (P = 0.006), oxidative stress evaluated by tert-butyl hydroperoxide-initiated chemiluminescence (P = 0.029), and advanced oxidation protein products (p=0.025) than those patients without IR. The multivariate analysis showed that disability was associated with IR evaluated by homeostasis model assessment of insulin resistance (p= 0.030) and adiposity evaluated by waist circumference (P ¼ 0.0179) and body mass index (p=0.003). |
| Sternberg et al, 2013  [8] | Retrospective chart review of 206 PwMS s vs 142 control patients with meningiomas and acoustic neuromas, non-inflammatory, non-autoimmune diseases of the brain | USA | Comparison of CV risk factors in MS and non-MS patients | MS patients had significantly higher total plasma cholesterol (p = )0.01, and plasma high density lipoprotein (p <0.001), but lower plasma glucose, (p <0.001), and systolic BP, (p = 0.001), than non-MS patients. In addition, MS patients had lower erythrocyte sedimentation rate and serum vitamin B12, but higher serum folic acid and vitamin D3 than non-MS patients (p≤0.05). A positive correlation was observed between plasma glucose and the extended disability status scale (EDSS), (p= 0.008), and between plasma glucose and the rate of clinical relapse, (p=0.001) |
| Rabadi et al, 2016  [114] | Retrospective review of the electronic charts of 124 veterans with MS who have been regularly followed in an MS clinic for 10 or more years Observational study  No control group | USA | Investigation of the effects of common chronic medical conditions (CMCs) on long-term disability (activity limitation) in veterans already diagnosed with MS. | Commonly encountered CMCs were increased BMI (61%), hyperlipidemia (78%), hypertension (65%), current smokers (47%), and arthritis/arthralgia (24%). Results suggest that the number of CMCs was not predictive of final Total Functional Independence Measure (TFIM) scores. Of the variables examined, only initial EDSS score was predictive of final TFIM scores (p=0.0003). The presence of CMCs did not affect the long-term disability in veterans diagnosed with MS (p>0.05), this was due mainly to CMCs being closely monitored and co-treated with other medical specialties.  No control group was present for comparison |
| Conway et al, 2017  [115] | Cohort study in 2083 PwMS  No control group | USA | Impact of hypertension, hyperlipidemia, diabetes mellitus, and obstructive  lung disease on disease course in MS patients | Prevalence of different comorbidities were diabetes (4.3%), hypertension (17.7%), hyperlipidemia (13.7%) and obstructive lung disease (7.3%). The 3-year longitudinal study revealed that all comorbidities tested except hyperlipidemia impacted clinical outcomes and a cumulative effect with multiple comorbidities was observed walking speed (p<0.02), performance scales (p<0.001) and PHQ (p<0.0001)  No control group was present for comparison |
| Kowalec et al, 2017  [12] | 2-year prospective multicenter cohort study in 885 PwMS  No control group | Canada | Cross-sectional assessment of comorbidities and relapses. Comorbidities were recorded using questionnaires, and relapses were captured from medical records at each visit. | Anxiety (40.2%), depression (21.1%), hypertension (17.7%), migraine (18.1%), and hyperlipidemia (11.9%) were the most prevalent comorbidities.  Migraine and hyperlipidemia were associated with increased relapse rate (adjusted rate  ratio 1.38; 95% CI 1.01–1.89 and 1.67, 95% CI 1.07–2.61, respectively)  No control group was present for comparison |
| Marrie et al, 2012  [116] | Case matched administrative data study in 4192 PwMS and 20,940 persons from the general population. The validation cohort included 430 PwMS | Canada | Prevalence of diabetes, hypertension, and hyperlipidemia in PwMS defined using hospital, physician, and prescription claims | The 2005 age-adjusted prevalence of diabetes was similar in the MS (7.62%) and general populations (8.31%; [PR] 0.91; 0.81–1.03). The age-adjusted prevalence did not differ for hypertension (MS: 20.8% versus general: 22.5% [PR 0.91; 0.78–1.06]), or hyperlipidemia (MS: 13.8% versus general: 15.2% [PR 0.90; 0.67–1.22]). The prevalence of all conditions rose in both populations over the study period |
| Tettey et al, 2016  [14] | Prospective cohort of 198 PwMS | Australia | Evaluation of specific doctor-diagnosed comorbidities in PwMS versus the 207 general population prevalence | The age-standardized prevalence of hypertension (21.21% vs 9.43%), dyslipidemia (14.65% vs 5.72%), asthma (18.69% vs9.93%), psoriasis (7.58% vs 2.29%), eczema (13.13% vs 0.92%) and anemia (12.63% vs 1.83%) were significantly higher in the MS cohort compared to that in the general Australian population (p≤0.001.  For relapse analyses, rheumatoid arthritis and anemia were associated with more than threefold (HR 3.70, 95% CI 1.80─7.58, p=0.001) and twofold (HR 2.04, 95% CI 1.11─3.74, p = 0.022) increased risk of subsequent relapse respectively. The prevalence of some comorbidities was higher in MS patients and associated with greater disability and relapse risk. |
| Pinhas-Hamiel et al, 2015  [117] | 130 PwMS compared to the general population | Israel | Prevalence of the metabolic syndrome (MetS) and its components in patients with long duration of MS and significant disability | Obesity [body mass index (BMI) ≥ 30 kg/m(2) ] was present in 18.5% and overweight (BMI 25.0-29.9 kg/m(2) ) in 34.6%. The prevalence of the MetS was 30% with no gender difference. Fifty-six percent had central obesity by waist circumference, 28% treated hypertension, 45.8% elevated blood pressure, 11% type 2 diabetes mellitus, 31.4% treated dyslipidemia, 28.8% elevated triglyceride levels and 31.4% had low HDL-C. MS patients with MetS were significantly older (59.0 ± 5.5 vs. 53.8 ± 5.5, P < 0.0001) and heavier (BMI 29.0 ± 6.9 vs. 25.1 ± 4.7, P = 0.0009). There were no differences between the groups in neurological disability by the EDSS (5.7 ± 1.0 vs. 5.4 ± 1.0), disease duration (18.4 ± 9.9 vs. 18.2 ± 10.2 years) and number of steroid courses received (6.6 ± 9.5 vs. 6.3 ± 8.4).Compared to the general population, adult disabled MS patients had lower rates of obesity and overweight, as assessed by BMI. Despite these reduced rates, the prevalence of the MetS was similar to the general population. Specifically, higher rates of increased waist circumference were found, suggesting that the lower BMI may be misleading in terms of health risk. |

### Table 9: Pulmonary disease

| Reference | Study type | Region | Measure | Outcome |
| --- | --- | --- | --- | --- |
| Manouchehrinia et a, 2015  [118] | Cohort study in 680 PwMS  No control group | UK | Investigation of asthma prevalence in MS patients and association with EDSS | There was no difference in the prevalence of asthma between the MS cohort and the English general population (OR: 0.89, 95% CI 0.68−1.17). No significant association between having asthma and the  risk of reaching EDSS scores 4.0 and 6.0 was found (HR: 1.29, 95% CI 0.93 to 1.77, and HR: 1.33, 95% CI 0.93−1.89, respectively) after controlling for confounders  No control group was present for comparison |
| Jick et al, 2015  [6] | Cohort study in 1713 PwMS.  No control group | UK | Assessment of lifetime incidence of comorbidity in PwMS | Following MS diagnosis, frequent comorbidities were infections (80 %), and depression (46 %). Incidence of chronic comorbidities recorded at any time in the database were: Chronic obstructive pulmonary disease (COPD) and asthma (20.0%) depression (45.8%) diabetes (5.4%) hypertension (14.8%) heart disease (3.9%) cancer (6.4%)  No control group was present for comparison. |
| Thormann et al, 2016  [119] | Cohort study and case-control study in same data with 8947 PwMS and 44,735 controls | Denmark | Comorbidity as exposure and outcome | Decreased occurrences of asthma and chronic obstructive pulmonary disease  (HR 0.80 (95% CI 0.75-0.86, p<0.00025) and overall cancer (HR 0.88 (95% CI 0.81-0.95, ,p=0.0005)) among MS-cases. |
| Hill et al, 2019  [120] | Population-based, cross-sectional study of electronic health records in 141,880 PwMS and 56,416,790 non-MS controls | USA | Investigation of Health record data for prevalence of asthma in PwMS | Adjusting for age and gender, asthma was three times more common in MS. The crude prevalence of asthma was 2.48 (95% CI: 2.45−2.52; p< <0.0001) times higher in the MS cohort than the non-MS cohort  (16.5% versus 6.7%, respectively) and after standardization, the age and gender adjusted PR of asthma was 2.97 (95% CI: 2.96−2.97; p<0.0001) |
| Zivadinov et al, 2016  [91] | Retrospective chart review in 815 PwMS, 241 with comorbidities and 574 without comorbidities | USA | Determined the association of comorbidities with MR imaging disease severity outcomes in MS | Two hundred forty-one (29.6%) study subjects presented with comorbidities. Thyroid disease had the highest frequency (n = 97, 11.9%), followed by asthma (n = 41, 5%), type 2 diabetes mellitus (n = 40, 4.9%), psoriasis (n = 33, 4%), and rheumatoid arthritis (n = 22, 2.7%). The presence of comorbidities in patients with MS is associated with brain injury (reduced brain volume) on MR imaging (p<0.05). Psoriasis, thyroid disease, and type 2 diabetes mellitus comorbidities were associated with more severe nonconventional MR imaging outcomes. |
| Conway et al, 2017  [115] | Cohort study in 2083 PwMS  No control group | USA | Impact of hypertension, hyperlipidemia, diabetes mellitus, and obstructive  lung disease on disease course in MS patients | Prevalence of different comorbidities were diabetes (4.3%), hypertension (17.7%), hyperlipidemia (13.7%) and obstructive lung disease (7.3%). The 3-year longitudinal study revealed that all comorbidities tested except hyperlipidemia impacted clinical outcomes and a cumulative effect with multiple comorbidities was observed walking speed (p<0.02), performance scales (p<0.001) and PHQ (p<0.0001)  No control group was present for comparison |
| Marrie et al, 2016  [13] | Population-based study of 23,382 PwMS and 116,638 matches from the general population | Canada | Prevalence of comorbidity in PwMS from administrative health data from 4 Canadian provinces, including British Columbia, Manitoba, Quebec, and Nova Scotia. | Crude prevalence of comorbidity in MS at diagnosis compared to matched controls. Hypertension (15.2% vs 12.9%, RR 1.17 (95% CI 1.13─1.21, p<,0.0001); diabetes (5.69% vs 4.86%, RR 1.17 (95% CI 1.10─1.24, p<0.0001); IHD (6.50% vs 5.01, RR 1.30 (95% CI 1.23─1.37, p<,0.0001); fibromyalgia (1.31% vs 0.46%, RR 2.87, 95% CI 2.49 ─3.30, p<0.0001); IBD (0.56% vs 0.30%, RR 1.68, 95% CI 1.38─2.04 p<0.0001). Chronic lung disease (12.1% vs 9.14%, RR 1.34, 95% CI 1.29─1.39); epilepsy (1.93 vs 0.89%, RR 2.18, 95% CI 1.95, 2.43, p<0.0001; depression (19.1% vs 9.38%, RR 2.04, 95% CI 1.97─2.10, p< 0.0001); Anxiety (11.1% vs 6.89%, RR 1.61, 95 CI% 1.54─1.68, p<0.0001; bipolar disorder (3.15% vs 1.69%, RR 1.86, 95% CI 1.71─2.02 p<0.0001; schizophrenia (1.07% vs 0.81%, RR 1.32, 95% CI 1.15─1.52, p<0.0001) in PwMS vs control population, respectively. |
| Marrie et al, 2016  [121] | Matched cohort study of 44,452 PwMS and 220,849 control subjects | Canada | Incidence and prevalence of CLD, including asthma and chronic obstructive pulmonary disease | Among persons aged 20-44 years the average annual incidence of CLD was higher in the MS population than in the matched population (RR 1.15; 95% CI: 1.02-1.30), but did not differ between populations for those aged ≥45 years. |
| Farez et al, 2014  [95] | Case-controlled study with 211 PwMS and 211 control subjects | Argentina | Prevalence of AID in MS compared with controls | 28% of MS patients reported at least one autoimmune comorbidity, with no significant differences with respect to healthy controls (32%, 𝜒2 0.45). Most frequently AIS in MR was thyroid disease (9%), asthma (6%), atopic dermatitis (5%), and type I diabetes (3%). There were no significant differences in autoimmune disease prevalence in PwMS with respect to controls. The presence of one or more autoimmune disorders did not increase risk of MS (OR 0.85, 95% CI 0.6–1.3) |
| Tettey et al, 2016  [14] | Prospective cohort of 198 PwMS | Australia | Evaluation of specific doctor-diagnosed comorbidities in PwMS versus the 207 general population prevalence | The age-standardized prevalence of hypertension (21.21% vs 9.43%), dyslipidemia (14.65% vs 5.72%), asthma (18.69% vs9.93%), psoriasis (7.58% vs 2.29%), eczema (13.13% vs 0.92%) and anemia (12.63% vs 1.83%) were significantly higher in the MS cohort compared to that in the general Australian population (p≤0.001.  For relapse analyses, rheumatoid arthritis and anemia were associated with more than threefold (HR 3.70, 95% CI 1.80─7.58, p=0.001) and twofold (HR 2.04, 95% CI 1.11─3.74, p = 0.022) increased risk of subsequent relapse respectively. The prevalence of some comorbidities was higher in MS patients and associated with greater disability and relapse risk. |
| Kang et al, 2010  [16] | Cohort study in 898 PwMS and 4490 control subjects | Taiwan | Evaluation of 30 comorbid medical conditions from insurance claims | PwMS were more likely to have systemic lupus erythematosus (OR = 26.9, 95% CI 10.3−70.3), depression (OR = 6.9, 95% CI 5.3─8.9), peripheral vascular disorders (OR = 6.6, 95% CI 4.0−11.0), deficiency anemias (OR = 4.9, 95% CI 2.8−8.7), rheumatoid arthritis (OR = 4.8, 95% CI 2.9−8.1) and fluid and electrolyte disorders (OR = 4.8, 95% CI 2.8−8.3) than the matched controls. Patients with MS had higher risk of multiple medical comorbidities compared to a matched control group in an ethnic Chinese population. |

### Abbreviations:

| AF, atrial fibrillation  AID, autoimmune disease  AMI, acute myocardial infarct  BDI, Becks depression index  BRB-N, brief Repeatable Battery of Neuropsychological tests  CES-D, The Center for Epidemiologic Studies - depression scale  CI, confidence interval CLD, chronic lung disease  CV, cardiovascular  CVD, cardiovascular disease  DAI, diffuse axonal injury  DMT, disease-modifying treatment  EDSS, Expanded Disability Status Scale  ESS, Epworth sleepiness scale  FSS, Kurtzke Functional Systems Scores  GA  GLTEQ, Godin Leisure-Time Exercise Questionnaire  HADS, hospital anxiety and depression scale  HADS-A, hospital anxiety and depression scale-anxiety  HADS-D, hospital anxiety and depression scale- depression  HF, heart failure  HRQoL, health-related quality of life  ICD, International classification  ICHD-2, International Classification of Headache Disorders  IFN-b  IHD, ischemic heart disease  IR, insulin resistance  IRLSSG, International Restless Legs Syndrome Study Group  IRR, incidence rate ratio  IGT, impaired glucose tolerance  NRS, numeric rating scale | IMSS, Isfahan MS society,  IPQ, Italian pain questionnaire  MACFIMS, minimal assessment of cognitive function in MS  MFIS, modified fatigue impact scale  MI, myocardial infarct  MIDAS, Migraine disability assessment  MS, MS  NTZ  OR, odds ratio  MSISQ-19, MS Intimacy and Sexuality Questionnaire-19  NPSI, Neuropathic Pain Symptom Inventory  PedsQL™, Pediatric Quality of Life Inventory  PR, prevalence ratio  PROMIS, Patient-Reported Outcomes Measurement Information System  PSQI, Pittsburgh sleep quality index  QoL, quality of life  RA, rheumatoid arthritis  RBD, REM sleep behavior disorder  RLS, restless leg syndrome  RR, risk ratio  SDMT, symbol digit modalities test  SIR, speciﬁc standardized incidence ratios  SPART, spatial recall test  SS, Sjogren's syndrome  SRT, scapular retraction test  STAI, State-Trait Anxiety Inventory  TAS, Toronto alexithymia scale  Trail Making Test, TMT  VAS, visual analogue scale  VTE, venous thromboembolism |
| --- | --- |

### References

1. Roshanisefat H, Bahmanyar S, Hillert J, Olsson T, Montgomery S (2014) Multiple sclerosis clinical course and cardiovascular disease risk - Swedish cohort study. Eur J Neurol 21 (11):1353-e1388. doi:10.1111/ene.12518

2. Jadidi E, Mohammadi M, Moradi T (2013) High risk of cardiovascular diseases after diagnosis of multiple sclerosis. Mult Scler 19 (10):1336-1340. doi:10.1177/1352458513475833

3. Christiansen CF, Christensen S, Farkas DK, Miret M, Sorensen HT, Pedersen L (2010) Risk of arterial cardiovascular diseases in patients with multiple sclerosis: a population-based cohort study. Neuroepidemiology 35 (4):267-274. doi:10.1159/000320245

4. Koudriavtseva T, Renna R, Plantone D, Mandoj C, Piattella MC, Giannarelli D (2015) Association between anemia and multiple sclerosis. Eur Neurol 73 (3-4):233-237. doi:10.1159/000381212

5. Peeters PJ, Bazelier MT, Uitdehaag BM, Leufkens HG, De Bruin ML, de Vries F (2014) The risk of venous thromboembolism in patients with multiple sclerosis: the Clinical Practice Research Datalink. J Thromb Haemost 12 (4):444-451. doi:10.1111/jth.12523

6. Jick SS, Li L, Falcone GJ, Vassilev ZP, Wallander MA (2015) Epidemiology of multiple sclerosis: results from a large observational study in the UK. J Neurol 262 (9):2033-2041. doi:10.1007/s00415-015-7796-2

7. Sternberg Z, Leung C, Sternberg D, Yu J, Hojnacki D (2014) Disease modifying therapies modulate cardiovascular risk factors in patients with multiple sclerosis. Cardiovasc Ther 32 (2):33-39. doi:10.1111/1755-5922.12049

8. Sternberg Z, Leung C, Sternberg D, Li F, Karmon Y, Chadha K, Levy E (2013) The prevalence of the classical and non-classical cardiovascular risk factors in multiple sclerosis patients. CNS Neurol Disord Drug Targets 12 (1):104-111. doi:10.2174/1871527311312010016

9. Kappus N, Weinstock-Guttman B, Hagemeier J, Kennedy C, Melia R, Carl E, Ramasamy DP, Cherneva M, Durfee J, Bergsland N, Dwyer MG, Kolb C, Hojnacki D, Ramanathan M, Zivadinov R (2016) Cardiovascular risk factors are associated with increased lesion burden and brain atrophy in multiple sclerosis. J Neurol Neurosurg Psychiatry 87 (2):181-187. doi:10.1136/jnnp-2014-310051

10. Marrie RA, Garland A, Schaffer SA, Fransoo R, Leung S, Yogendran M, Kingwell E, Tremlett H (2019) Traditional risk factors may not explain increased incidence of myocardial infarction in MS. Neurology 92 (14):e1624-e1633. doi:10.1212/WNL.0000000000007251

11. Marrie RA, Yu BN, Leung S, Elliott L, Caetano P, Warren S, Wolfson C, Patten SB, Svenson LW, Tremlett H, Fisk J, Blanchard JF (2013) Prevalence and incidence of ischemic heart disease in multiple sclerosis: A population-based validation study. Mult Scler Relat Disord 2 (4):355-361. doi:10.1016/j.msard.2013.03.001

12. Kowalec K, McKay KA, Patten SB, Fisk JD, Evans C, Tremlett H, Marrie RA, Epidemiology CTi, Impact of Comorbidity on Multiple S (2017) Comorbidity increases the risk of relapse in multiple sclerosis: A prospective study. Neurology 89 (24):2455-2461. doi:10.1212/WNL.0000000000004716

13. Marrie RA, Patten SB, Tremlett H, Wolfson C, Warren S, Svenson LW, Jette N, Fisk J, Epidemiology CTit, Impact of Comorbidity on Multiple S (2016) Sex differences in comorbidity at diagnosis of multiple sclerosis: A population-based study. Neurology 86 (14):1279-1286. doi:10.1212/WNL.0000000000002481

14. Tettey P, Siejka D, Simpson S, Jr., Taylor B, Blizzard L, Ponsonby AL, Dwyer T, van der Mei I (2016) Frequency of Comorbidities and Their Association with Clinical Disability and Relapse in Multiple Sclerosis. Neuroepidemiology 46 (2):106-113. doi:10.1159/000442203

15. Tseng CH, Huang WS, Lin CL, Chang YJ (2015) Increased risk of ischaemic stroke among patients with multiple sclerosis. Eur J Neurol 22 (3):500-506. doi:10.1111/ene.12598

16. Kang JH, Chen YH, Lin HC (2010) Comorbidities amongst patients with multiple sclerosis: a population-based controlled study. Eur J Neurol 17 (9):1215-1219. doi:10.1111/j.1468-1331.2010.02971.x

17. Ben Ari Shevil E, Johansson S, Ytterberg C, Bergstrom J, von Koch L (2014) How are cognitive impairment, fatigue and signs of depression related to participation in daily life among persons with multiple sclerosis? Disabil Rehabil 36 (23):2012-2018. doi:10.3109/09638288.2014.887797

18. O'Connell K, Tubridy N, Hutchinson M, McGuigan C (2017) Incidence of multiple sclerosis in the Republic of Ireland: A prospective population-based study. Mult Scler Relat Disord 13:75-80. doi:10.1016/j.msard.2017.02.010

19. Solaro C, Trabucco E, Signori A, Martinelli V, Radaelli M, Centonze D, Rossi S, Grasso MG, Clemenzi A, Bonavita S, D'Ambrosio A, Patti F, D'Amico E, Cruccu G, Truini A (2016) Depressive Symptoms Correlate with Disability and Disease Course in Multiple Sclerosis Patients: An Italian Multi-Center Study Using the Beck Depression Inventory. PLoS One 11 (9):e0160261. doi:10.1371/journal.pone.0160261

20. Mattioli F, Bellomi F, Stampatori C, Parrinello G, Capra R (2011) Depression, disability and cognitive impairment in multiple sclerosis: a cross sectional Italian study. Neurol Sci 32 (5):825-832. doi:10.1007/s10072-011-0624-2

21. Rossi S, Studer V, Motta C, Polidoro S, Perugini J, Macchiarulo G, Giovannetti AM, Pareja-Gutierrez L, Calo A, Colonna I, Furlan R, Martino G, Centonze D (2017) Neuroinflammation drives anxiety and depression in relapsing-remitting multiple sclerosis. Neurology 89 (13):1338-1347. doi:10.1212/WNL.0000000000004411

22. Garfield AC, Lincoln NB (2012) Factors affecting anxiety in multiple sclerosis. Disabil Rehabil 34 (24):2047-2052. doi:10.3109/09638288.2012.667503

23. Sicras-Mainar A, Ruiz-Beato E, Navarro-Artieda R, Maurino J (2017) Comorbidity and metabolic syndrome in patients with multiple sclerosis from Asturias and Catalonia, Spain. BMC Neurol 17 (1):134. doi:10.1186/s12883-017-0914-2

24. Schmidt S, Jostingmeyer P (2019) Depression, fatigue and disability are independently associated with quality of life in patients with multiple Sclerosis: Results of a cross-sectional study. Mult Scler Relat Disord 35:262-269. doi:10.1016/j.msard.2019.07.029

25. Kale N, Agaoglu J, Tanik O (2010) Neuropsychiatric manifestations in multiple sclerosis: correlation of fatigue and depression with disease progression. Neurol Res 32 (2):221-223. doi:10.1179/174313209X455664

26. Maier S, Buruian M, Maier A, Motataianu A, Voidazan S, Bajko Z, Balasa R (2016) The determinants of depression in a Romanian cohort of multiple sclerosis patients. Acta Neurol Belg 116 (2):135-143. doi:10.1007/s13760-015-0547-4

27. Hoang H, Laursen B, Stenager EN, Stenager E (2016) Psychiatric co-morbidity in multiple sclerosis: The risk of depression and anxiety before and after MS diagnosis. Mult Scler 22 (3):347-353. doi:10.1177/1352458515588973

28. Persson R, Lee S, Yood MU, Wagner M, Minton N, Niemcryk S, Lindholm A, Evans A, Jick S (2019) Multi-database study of multiple sclerosis: identification, validation and description of MS patients in two countries. J Neurol 266 (5):1095-1106. doi:10.1007/s00415-019-09238-8

29. Alschuler KN, Jensen MP, Ehde DM (2012) The association of depression with pain-related treatment utilization in patients with multiple sclerosis. Pain Med 13 (12):1648-1657. doi:10.1111/j.1526-4637.2012.01513.x

30. Newland P, Jensen MP, Budhathoki C, Lorenz R (2015) Secondary health conditions in individuals with multiple sclerosis: a cross-sectional web-based survey analysis. J Neurosci Nurs 47 (3):124-130. doi:10.1097/JNN.0000000000000130

31. Jun-O'Connell AH, Butala A, Morales IB, Henninger N, Deligiannidis KM, Byatt N, Ionete C (2017) The Prevalence of Bipolar Disorders and Association With Quality of Life in a Cohort of Patients With Multiple Sclerosis. J Neuropsychiatry Clin Neurosci 29 (1):45-51. doi:10.1176/appi.neuropsych.15120403

32. Burns MN, Nawacki E, Siddique J, Pelletier D, Mohr DC (2013) Prospective examination of anxiety and depression before and during confirmed and pseudoexacerbations in patients with multiple sclerosis. Psychosom Med 75 (1):76-82. doi:10.1097/PSY.0b013e3182757b2b

33. Edwards KA, Molton IR, Smith AE, Ehde DM, Bombardier CH, Battalio SL, Jensen MP (2016) Relative Importance of Baseline Pain, Fatigue, Sleep, and Physical Activity: Predicting Change in Depression in Adults With Multiple Sclerosis. Arch Phys Med Rehabil 97 (8):1309-1315. doi:10.1016/j.apmr.2016.02.025

34. Berzins SA, Bulloch AG, Burton JM, Dobson KS, Fick GH, Patten SB (2017) Determinants and incidence of depression in multiple sclerosis: A prospective cohort study. J Psychosom Res 99:169-176. doi:10.1016/j.jpsychores.2017.06.012

35. Gill S, Santo J, Blair M, Morrow SA (2019) Depressive Symptoms Are Associated With More Negative Functional Outcomes Than Anxiety Symptoms in Persons With Multiple Sclerosis. J Neuropsychiatry Clin Neurosci 31 (1):37-42. doi:10.1176/appi.neuropsych.18010011

36. Theaudin M, Romero K, Feinstein A (2016) In multiple sclerosis anxiety, not depression, is related to gender. Mult Scler 22 (2):239-244. doi:10.1177/1352458515588582

37. Koch MW, Patten S, Berzins S, Zhornitsky S, Greenfield J, Wall W, Metz LM (2015) Depression in multiple sclerosis: a long-term longitudinal study. Mult Scler 21 (1):76-82. doi:10.1177/1352458514536086

38. Viner R, Fiest KM, Bulloch AG, Williams JV, Lavorato DH, Berzins S, Jette N, Metz LM, Patten SB (2014) Point prevalence and correlates of depression in a national community sample with multiple sclerosis. Gen Hosp Psychiatry 36 (3):352-354. doi:10.1016/j.genhosppsych.2013.12.011

39. Pham T, Jette N, Bulloch AGM, Burton JM, Wiebe S, Patten SB (2018) The prevalence of anxiety and associated factors in persons with multiple sclerosis. Mult Scler Relat Disord 19:35-39. doi:10.1016/j.msard.2017.11.003

40. Marrie RA, Walld R, Bolton JM, Sareen J, Walker JR, Patten SB, Singer A, Lix LM, Hitchon CA, El-Gabalawy R, Katz A, Fisk JD, Bernstein CN, Burden CTiDt, Managing the Effects of Psychiatric Comorbidity in Chronic Immunoinflammatory D (2017) Estimating annual prevalence of depression and anxiety disorder in multiple sclerosis using administrative data. BMC Res Notes 10 (1):619. doi:10.1186/s13104-017-2958-1

41. Morrow SA, Rosehart H, Pantazopoulos K (2016) Anxiety and Depressive Symptoms Are Associated With Worse Performance on Objective Cognitive Tests in MS. J Neuropsychiatry Clin Neurosci 28 (2):118-123. doi:10.1176/appi.neuropsych.15070167

42. McKay KA, Tremlett H, Fisk JD, Patten SB, Fiest K, Berrigan L, Marrie RA, Epidemiology CTit, Impact of Comorbidity on Multiple S (2016) Adverse health behaviours are associated with depression and anxiety in multiple sclerosis: A prospective multisite study. Mult Scler 22 (5):685-693. doi:10.1177/1352458515599073

43. McKay KA, Tremlett H, Fisk JD, Zhang T, Patten SB, Kastrukoff L, Campbell T, Marrie RA, Epidemiology CTit, Impact of Comorbidity on Multiple S (2018) Psychiatric comorbidity is associated with disability progression in multiple sclerosis. Neurology 90 (15):e1316-e1323. doi:10.1212/WNL.0000000000005302

44. de Cerqueira AC, Semionato de Andrade P, Godoy Barreiros JM, Teixeira AL, Nardi AE (2015) Psychiatric disorders in patients with multiple sclerosis. Compr Psychiatry 63:10-14. doi:10.1016/j.comppsych.2015.08.001

45. Wood B, van der Mei IA, Ponsonby AL, Pittas F, Quinn S, Dwyer T, Lucas RM, Taylor BV (2013) Prevalence and concurrence of anxiety, depression and fatigue over time in multiple sclerosis. Mult Scler 19 (2):217-224. doi:10.1177/1352458512450351

46. Taylor KL, Hadgkiss EJ, Jelinek GA, Weiland TJ, Pereira NG, Marck CH, van der Meer DM (2014) Lifestyle factors, demographics and medications associated with depression risk in an international sample of people with multiple sclerosis. BMC Psychiatry 14:327. doi:10.1186/s12888-014-0327-3

47. Knippenberg S, Damoiseaux J, Bol Y, Hupperts R, Taylor BV, Ponsonby AL, Dwyer T, Simpson S, van der Mei IA (2014) Higher levels of reported sun exposure, and not vitamin D status, are associated with less depressive symptoms and fatigue in multiple sclerosis. Acta Neurol Scand 129 (2):123-131. doi:10.1111/ane.12155

48. Simpson S, Jr., Taylor KL, Jelinek GA, De Livera AM, Brown CR, O'Kearney E, Neate SL, Bevens W, Weiland TJ (2019) Associations of demographic and clinical factors with depression over 2.5-years in an international prospective cohort of people living with MS. Mult Scler Relat Disord 30:165-175. doi:10.1016/j.msard.2019.02.014

49. Kalron A, Aloni R (2018) Contrasting relationship between depression, quantitative gait characteristics and self-report walking difficulties in people with multiple sclerosis. Mult Scler Relat Disord 19:1-5. doi:10.1016/j.msard.2017.10.012

50. Askari F, Ghajarzadeh M, Mohammadifar M, Azimi A, Sahraian MA, Owji M (2014) Anxiety in patients with multiple sclerosis: association with disability, depression, disease type and sex. Acta Med Iran 52 (12):889-892

51. Seyed Saadat SM, Hosseininezhad M, Bakhshayesh B, Seyed Saadat SN, Nabizadeh SP (2014) Prevalence and predictors of depression in Iranian patients with multiple sclerosis: a population-based study. Neurol Sci 35 (5):735-740. doi:10.1007/s10072-013-1593-4

52. Schiess N, Huether K, Holroyd KB, Aziz F, Emam E, Shahrour T, Szolics M, Alsaadi T (2019) Multiple Sclerosis, Anxiety, and Depression in the United Arab Emirates: Does Social Stigma Prevent Treatment? Int J MS Care 21 (1):29-34. doi:10.7224/1537-2073.2017-041

53. Alsaadi T, El Hammasi K, Shahrour TM, Shakra M, Turkawi L, Mudhafar A, Diab L, Raoof M (2015) Prevalence of Depression and Anxiety among Patients with Multiple Sclerosis Attending the MS Clinic at Sheikh Khalifa Medical City, UAE: Cross-Sectional Study. Mult Scler Int 2015:487159. doi:10.1155/2015/487159

54. Al-Asmi A, Al-Rawahi S, Al-Moqbali ZS, Al-Farsi Y, Essa MM, El-Bouri M, Koshy RP, Gujjar AR, Jacob PC, Al-Hodar A, Al Adawi S (2015) Magnitude and concurrence of anxiety and depression among attendees with multiple sclerosis at a tertiary care Hospital in Oman. BMC Neurol 15:131. doi:10.1186/s12883-015-0370-9

55. Gasparini S, Ferlazzo E, Ascoli M, Sueri C, Cianci V, Russo C, Pisani LR, Striano P, Elia M, Beghi E, Colica C, Aguglia U, Epilepsy Study Group of the Italian Neurological S (2017) Risk factors for unprovoked epileptic seizures in multiple sclerosis: a systematic review and meta-analysis. Neurol Sci 38 (3):399-406. doi:10.1007/s10072-016-2803-7

56. Krokki O, Bloigu R, Ansakorpi H, Reunanen M, Remes AM (2014) Neurological comorbidity and survival in multiple sclerosis. Mult Scler Relat Disord 3 (1):72-77. doi:10.1016/j.msard.2013.06.006

57. Burman J, Zelano J (2017) Epilepsy in multiple sclerosis: A nationwide population-based register study. Neurology 89 (24):2462-2468. doi:10.1212/WNL.0000000000004740

58. Martinez-Lapiscina EH, Ayuso T, Lacruz F, Gurtubay IG, Soriano G, Otano M, Bujanda M, Bacaicoa MC (2013) Cortico-juxtacortical involvement increases risk of epileptic seizures in multiple sclerosis. Acta Neurol Scand 128 (1):24-31. doi:10.1111/ane.12064

59. Benjaminsen E, Myhr KM, Alstadhaug KB (2017) The prevalence and characteristics of epilepsy in patients with multiple sclerosis in Nordland county, Norway. Seizure 52:131-135. doi:10.1016/j.seizure.2017.09.022

60. Allen AN, Seminog OO, Goldacre MJ (2013) Association between multiple sclerosis and epilepsy: large population-based record-linkage studies. BMC Neurol 13:189. doi:10.1186/1471-2377-13-189

61. Viveiros CD, Alvarenga RM (2010) Prevalence of epilepsy in a case series of multiple sclerosis patients. Arq Neuropsiquiatr 68 (5):731-736. doi:10.1590/s0004-282x2010000500011

62. Uribe-San-Martin R, Ciampi-Diaz E, Suarez-Hernandez F, Vasquez-Torres M, Godoy-Fernandez J, Carcamo-Rodriguez C (2014) Prevalence of epilepsy in a cohort of patients with multiple sclerosis. Seizure 23 (1):81-83. doi:10.1016/j.seizure.2013.09.008

63. Etemadifar M, Abtahi SH, Tabrizi N (2012) Epileptic seizures in early-onset multiple sclerosis. Arch Iran Med 15 (6):381-383. doi:012156/AIM.0014

64. Etemadifar M, Abtahi SH, Roomizadeh P (2013) Epileptic seizures in multiple sclerosis: a population-based survey in Iran. Acta Neurol Belg 113 (3):271-278. doi:10.1007/s13760-012-0146-6

65. Lebrato Hernandez L, Prieto Leon M, Cerda Fuentes NA, Ucles Sanchez AJ, Casado Chocan JL, Diaz Sanchez M (2019) Restless legs syndrome in patients with multiple sclerosis: evaluation of risk factors and clinical impact. Neurologia. doi:10.1016/j.nrl.2018.12.010

66. Bruno E, Nicoletti A, Messina S, Lo Fermo S, Raciti L, Quattrocchi G, Dibilio V, Paradisi V, Practitioners SG, Maimone D, Patti F, Zappia M (2015) Restless legs syndrome and multiple sclerosis: a population based case-control study in Catania, Sicily. Eur J Neurol 22 (6):1018-1021. doi:10.1111/ene.12409

67. Minar M, Petrlenicova D, Valkovic P (2017) Higher prevalence of restless legs syndrome/Willis-Ekbom disease in multiple sclerosis patients is related to spinal cord lesions. Mult Scler Relat Disord 12:54-58. doi:10.1016/j.msard.2016.12.013

68. Schurks M, Bussfeld P (2013) Multiple sclerosis and restless legs syndrome: a systematic review and meta-analysis. Eur J Neurol 20 (4):605-615. doi:10.1111/j.1468-1331.2012.03873.x

69. Sorgun MH, Aksun Z, Atalay YB, Yucesan C (2015) Restless legs syndrome in multiple sclerosis. Turk J Med Sci 45 (6):1268-1273. doi:10.3906/sag-1401-19

70. Carnero Contentti E, Lopez PA, Nadur D, Balbuena ME, Finkelstein AM, Tkachuk V (2019) Impact, Frequency, and Severity of Restless Legs Syndrome in Patients with Multiple Sclerosis in Argentina. Int J MS Care 21 (4):157-165. doi:10.7224/1537-2073.2018-009

71. Ning P, Hu F, Yang B, Shen Q, Zhao Q, Huang H, An R, Chen Y, Wang H, Yang X, Xu Y (2018) Systematic review and meta-analysis of observational studies to understand the prevalence of restless legs syndrome in multiple sclerosis: an update. Sleep Med 50:97-104. doi:10.1016/j.sleep.2018.05.039

72. Liu G, Feng X, Lan C, Zhu Z, Ma S, Guo Y, Xue R (2015) Restless leg syndrome and multiple sclerosis: a case-control study in China. Sleep Breath 19 (4):1355-1360. doi:10.1007/s11325-015-1201-3

73. Miri S, Rohani M, Sahraian MA, Zamani B, Shahidi GA, Sabet A, Moradi-Lakeh M, Bodaghabadi M (2013) Restless legs syndrome in Iranian patients with multiple sclerosis. Neurol Sci 34 (7):1105-1108. doi:10.1007/s10072-012-1186-7

74. Shaygannejad V, Ardestani PE, Ghasemi M, Meamar R (2013) Restless legs syndrome in Iranian multiple sclerosis patients: a case-control study. Int J Prev Med 4 (Suppl 2):S189-193

75. Pakpoor J, Handel AE, Giovannoni G, Dobson R, Ramagopalan SV (2012) Meta-analysis of the relationship between multiple sclerosis and migraine. PLoS One 7 (9):e45295. doi:10.1371/journal.pone.0045295

76. Bazelier MT, Mueller-Schotte S, Leufkens HG, Uitdehaag BM, van Staa T, de Vries F (2012) Risk of cataract and glaucoma in patients with multiple sclerosis. Mult Scler 18 (5):628-638. doi:10.1177/1352458511426737

77. Villani V, De Giglio L, Sette G, Pozzilli C, Salvetti M, Prosperini L (2012) Determinants of the severity of comorbid migraine in multiple sclerosis. Neurol Sci 33 (6):1345-1353. doi:10.1007/s10072-012-1119-5

78. Silva AM, Santos E, Moreira I, Bettencourt A, Coutinho E, Goncalves A, Pinto C, Montalban X, Cavaco S (2012) Olfactory dysfunction in multiple sclerosis: association with secondary progression. Mult Scler 18 (5):616-621. doi:10.1177/1352458511427156

79. Sahai-Srivastava S, Wang SL, Ugurlu C, Amezcua L (2016) Headaches in multiple sclerosis: Cross-sectional study of a multiethnic population. Clin Neurol Neurosurg 143:71-75. doi:10.1016/j.clineuro.2016.01.017

80. Lincoff NS, Buccilli A, Weinstock-Guttman B, Sieminski S, Gandhi S (2017) Is Multiple Sclerosis Associated With a Lower Intraocular Pressure? J Neuroophthalmol 37 (3):265-267. doi:10.1097/WNO.0000000000000520

81. Kister I, Caminero AB, Monteith TS, Soliman A, Bacon TE, Bacon JH, Kalina JT, Inglese M, Herbert J, Lipton RB (2010) Migraine is comorbid with multiple sclerosis and associated with a more symptomatic MS course. J Headache Pain 11 (5):417-425. doi:10.1007/s10194-010-0237-9

82. Marrie RA, Yu BN, Leung S, Elliott L, Warren S, Wolfson C, Tremlett H, Blanchard J, Fisk JD, Epidemiology CTit, Impact of Comorbidity in Multiple S (2012) The incidence and prevalence of fibromyalgia are higher in multiple sclerosis than the general population: A population-based study. Mult Scler Relat Disord 1 (4):162-167. doi:10.1016/j.msard.2012.06.001

83. Jordy SS, Starzewski AJ, Macedo FA, Manica GR, Tilbery CP, Carabetta EG (2016) Olfactory alterations in patients with multiple sclerosis. Arq Neuropsiquiatr 74 (9):697-700. doi:10.1590/0004-282X20160128

84. Liu CY, Tung TH, Lee CY, Chang KH, Wang SH, Chi CC (2019) Association of Multiple Sclerosis with Psoriasis: A Systematic Review and Meta-Analysis of Observational Studies. Am J Clin Dermatol 20 (2):201-208. doi:10.1007/s40257-018-0399-9

85. Lorefice L, Fenu G, Pitzalis R, Scalas G, Frau J, Coghe G, Musu L, Sechi V, Barracciu MA, Marrosu MG, Cocco E (2018) Autoimmune comorbidities in multiple sclerosis: what is the influence on brain volumes? A case-control MRI study. J Neurol 265 (5):1096-1101. doi:10.1007/s00415-018-8811-1

86. Annunziata P, De Santi L, Di Rezze S, Millefiorini E, Capello E, Mancardi G, De Riz M, Scarpini E, Vecchio R, Patti F (2011) Clinical features of Sjogren's syndrome in patients with multiple sclerosis. Acta Neurol Scand 124 (2):109-114. doi:10.1111/j.1600-0404.2010.01428.x

87. Fanouriakis A, Mastorodemos V, Pamfil C, Papadaki E, Sidiropoulos P, Plaitakis A, Amoiridis G, Bertsias G, Boumpas DT (2014) Coexistence of systemic lupus erythematosus and multiple sclerosis: prevalence, clinical characteristics, and natural history. Semin Arthritis Rheum 43 (6):751-758. doi:10.1016/j.semarthrit.2013.11.007

88. Deretzi G, Kountouras J, Koutlas E, Zavos C, Polyzos S, Rudolf J, Grigoriadis N, Gavalas E, Boziki M, Tsiptsios I (2010) Familial prevalence of autoimmune disorders in multiple sclerosis in Northern Greece. Mult Scler 16 (9):1091-1101. doi:10.1177/1352458510375708

89. Egeberg A, Mallbris L, Gislason GH, Skov L, Hansen PR (2015) Risk of Multiple Sclerosis in Patients with Psoriasis: A Danish Nationwide Cohort Study. J Invest Dermatol. doi:10.1038/jid.2015.350

90. Chouhfeh L, Kavak KS, Teter BE, Weinstock-Guttman B (2015) Disease modifying therapies use associated with comorbid autoimmune diseases in multiple sclerosis patients. Mult Scler Relat Disord 4 (3):228-233. doi:10.1016/j.msard.2015.02.004

91. Zivadinov R, Raj B, Ramanathan M, Teter B, Durfee J, Dwyer MG, Bergsland N, Kolb C, Hojnacki D, Benedict RH, Weinstock-Guttman B (2016) Autoimmune Comorbidities Are Associated with Brain Injury in Multiple Sclerosis. AJNR Am J Neuroradiol 37 (6):1010-1016. doi:10.3174/ajnr.A4681

92. Guido N, Cices A, Ibler E, Huynh T, Majewski S, Sable K, Rangel SM, West DP, Laumann AE, Nardone B (2017) Multiple sclerosis association with psoriasis: a large U.S. population, single centre, retrospective cross-sectional study. J Eur Acad Dermatol Venereol 31 (9):e397-e398. doi:10.1111/jdv.14205

93. Marrie RA, Patten SB, Tremlett H, Wolfson C, Leung S, Fisk JD (2017) Increased incidence and prevalence of psoriasis in multiple sclerosis. Mult Scler Relat Disord 13:81-86. doi:10.1016/j.msard.2017.02.012

94. Marrie RA, Yu BN, Leung S, Elliott L, Warren S, Wolfson C, Tremlett H, Fisk J, Blanchard J (2012) The incidence and prevalence of thyroid disease do not differ in the multiple sclerosis and general populations: a validation study using administrative data. Neuroepidemiology 39 (2):135-142. doi:10.1159/000339757

95. Farez MF, Balbuena Aguirre ME, Varela F, Kohler AA, Correale J (2014) Autoimmune disease prevalence in a multiple sclerosis cohort in Argentina. Mult Scler Int 2014:828162. doi:10.1155/2014/828162

96. Tseng CC, Chang SJ, Tsai WC, Ou TT, Wu CC, Sung WY, Hsieh MC, Yen JH (2016) Increased incidence of rheumatoid arthritis in multiple sclerosis: A nationwide cohort study. Medicine (Baltimore) 95 (26):e3999. doi:10.1097/MD.0000000000003999

97. Miron G, Gurevich M, Baum S, Achiron A, Barzilai A (2017) Psoriasis comorbidity affects multiple sclerosis neurological progression: a retrospective case - control analysis. J Eur Acad Dermatol Venereol 31 (12):2055-2061. doi:10.1111/jdv.14403

98. Fellner A, Dano M, Regev K, Mosek A, Karni A (2014) Multiple sclerosis is associated with psoriasis. A case-control study. J Neurol Sci 338 (1-2):226-228. doi:10.1016/j.jns.2014.01.003

99. Hongell K, Kurki S, Sumelahti ML, Soilu-Hanninen M (2019) Risk of cancer among Finnish multiple sclerosis patients. Mult Scler Relat Disord 35:221-227. doi:10.1016/j.msard.2019.08.005

100. Norgaard M, Veres K, Didden EM, Wormser D, Magyari M (2019) Multiple sclerosis and cancer incidence: A Danish nationwide cohort study. Mult Scler Relat Disord 28:81-85. doi:10.1016/j.msard.2018.12.014

101. Handel AE, Ramagopalan SV (2010) Multiple sclerosis and risk of cancer: a meta-analysis. J Neurol Neurosurg Psychiatry 81 (12):1413-1414. doi:10.1136/jnnp.2009.195776

102. Moisset X, Perie M, Pereira B, Dumont E, Lebrun-Frenay C, Lesage FX, Dutheil F, Taithe F, Clavelou P (2017) Decreased prevalence of cancer in patients with multiple sclerosis: A case-control study. PLoS One 12 (11):e0188120. doi:10.1371/journal.pone.0188120

103. Lebrun C, Vermersch P, Brassat D, Defer G, Rumbach L, Clavelou P, Debouverie M, de Seze J, Wiertlevsky S, Heinzlef O, Tourbah A, Fromont A, Frenay M (2011) Cancer and multiple sclerosis in the era of disease-modifying treatments. J Neurol 258 (7):1304-1311. doi:10.1007/s00415-011-5929-9

104. D'Amico E, Chisari CG, Arena S, Zanghi A, Toscano S, Lo Fermo S, Maimone D, Castaing M, Sciacca S, Zappia M, Patti F (2019) Cancer Risk and Multiple Sclerosis: Evidence From a Large Italian Cohort. Front Neurol 10:337. doi:10.3389/fneur.2019.00337

105. Ragonese P, Aridon P, Vazzoler G, Mazzola MA, Lo Re V, Lo Re M, Realmuto S, Alessi S, D'Amelio M, Savettieri G, Salemi G (2017) Association between multiple sclerosis, cancer risk, and immunosuppressant treatment: a cohort study. BMC Neurol 17 (1):155. doi:10.1186/s12883-017-0932-0

106. Hajiebrahimi M, Montgomery S, Burkill S, Bahmanyar S (2016) Risk of Premenopausal and Postmenopausal Breast Cancer among Multiple Sclerosis Patients. PLoS One 11 (10):e0165027. doi:10.1371/journal.pone.0165027

107. Kingwell E, Evans C, Zhu F, Oger J, Hashimoto S, Tremlett H (2014) Assessment of cancer risk with beta-interferon treatment for multiple sclerosis. J Neurol Neurosurg Psychiatry 85 (10):1096-1102. doi:10.1136/jnnp-2013-307238

108. Kingwell E, van der Kop M, Zhao Y, Shirani A, Zhu F, Oger J, Tremlett H (2012) Relative mortality and survival in multiple sclerosis: findings from British Columbia, Canada. J Neurol Neurosurg Psychiatry 83 (1):61-66. doi:10.1136/jnnp-2011-300616

109. Gaindh D, Kavak KS, Teter B, Vaughn CB, Cookfair D, Hahn T, Weinstock-Guttman B, New York State Multiple Sclerosis C (2016) Decreased risk of cancer in multiple sclerosis patients and analysis of the effect of disease modifying therapies on cancer risk. J Neurol Sci 370:13-17. doi:10.1016/j.jns.2016.09.005

110. Etemadifar M, Jahanbani-Ardakani H, Ghaffari S, Fereidan-Esfahani M, Changaei H, Aghadoost N, Jahanbani Ardakani A, Moradkhani N (2017) Cancer risk among patients with multiple sclerosis: A cohort study in Isfahan, Iran. Caspian J Intern Med 8 (3):172-177. doi:10.22088/cjim.8.3.172

111. Sun LM, Lin CL, Chung CJ, Liang JA, Sung FC, Kao CH (2014) Increased breast cancer risk for patients with multiple sclerosis: a nationwide population-based cohort study. Eur J Neurol 21 (2):238-244. doi:10.1111/ene.12267

112. Wens I, Dalgas U, Deckx N, Cools N, Eijnde BO (2014) Does multiple sclerosis affect glucose tolerance? Mult Scler 20 (9):1273-1276. doi:10.1177/1352458513515957

113. Oliveira SR, Simao AN, Kallaur AP, de Almeida ER, Morimoto HK, Lopes J, Dichi I, Kaimen-Maciel DR, Reiche EM (2014) Disability in patients with multiple sclerosis: influence of insulin resistance, adiposity, and oxidative stress. Nutrition 30 (3):268-273. doi:10.1016/j.nut.2013.08.001

114. Rabadi MH, Aston CE (2016) Effect of Chronic Medical Conditions in Veterans with Multiple Sclerosis on Long-Term Disability. Med Sci Monit 22:2768-2774. doi:10.12659/msm.900367

115. Conway DS, Thompson NR, Cohen JA (2017) Influence of hypertension, diabetes, hyperlipidemia, and obstructive lung disease on multiple sclerosis disease course. Mult Scler 23 (2):277-285. doi:10.1177/1352458516650512

116. Marrie RA, Yu BN, Leung S, Elliott L, Caetano P, Warren S, Wolfson C, Patten SB, Svenson LW, Tremlett H, Fisk J, Blanchard JF, Epidemiology CTi, Impact of Comorbidity on Multiple S (2012) Rising prevalence of vascular comorbidities in multiple sclerosis: validation of administrative definitions for diabetes, hypertension, and hyperlipidemia. Mult Scler 18 (9):1310-1319. doi:10.1177/1352458512437814

117. Pinhas-Hamiel O, Livne M, Harari G, Achiron A (2015) Prevalence of overweight, obesity and metabolic syndrome components in multiple sclerosis patients with significant disability. Eur J Neurol 22 (9):1275-1279. doi:10.1111/ene.12738

118. Manouchehrinia A, Edwards LJ, Roshanisefat H, Tench CR, Constantinescu CS (2015) Multiple sclerosis course and clinical outcomes in patients with comorbid asthma: a survey study. BMJ Open 5 (5):e007806. doi:10.1136/bmjopen-2015-007806

119. Thormann A, Koch-Henriksen N, Laursen B, Sorensen PS, Magyari M (2016) Inverse comorbidity in multiple sclerosis: Findings in a complete nationwide cohort. Mult Scler Relat Disord 10:181-186. doi:10.1016/j.msard.2016.10.008

120. Hill E, Abboud H, Briggs FBS (2019) Prevalence of asthma in multiple sclerosis: A United States population-based study. Mult Scler Relat Disord 28:69-74. doi:10.1016/j.msard.2018.12.012

121. Marrie RA, Patten S, Tremlett H, Svenson LW, Wolfson C, Yu BN, Elliott L, Profetto-McGrath J, Warren S, Leung S, Jette N, Bhan V, Fisk JD (2016) Chronic lung disease and multiple sclerosis: Incidence, prevalence, and temporal trends. Mult Scler Relat Disord 8:86-92. doi:10.1016/j.msard.2016.05.009
